# Supplementary material for: A general large-scale synthesis approach for crystalline porous materials
Source: Nat Commun. 2023 Nov 2;14:7022. doi: 10.1038/s41467-023-42833-y (PMC10622494; doi:10.1038/s41467-023-42833-y)
Supplement: Supplementary file 1 — Supplementary Information [file 41467_2023_42833_MOESM1_ESM.pdf]

Supplementary Information

A General Large-Scale Synthesis Approach for Crystalline Porous Materials

Xiongli Liu,[1] An Wang,[2] Chunping Wang,[2] Jinli Li,[1] Zhiyuan Zhang,[1] Abdullah M. Al-Enizi,[3] Ayman Nafady,[3] Feng Shui,[1] Zifeng You,[1] Baiyan Li,*[,1] Yangbing Wen,*[,2] and Shengqian Ma*[,4]

[1]School of Materials Science and Engineering, National Institute for Advanced Materials, TKL of Metal and Molecule-Based Material Chemistry, School of Materials Science and Engineering & Smart Sensing Interdisciplinary Science Center, Nankai University, Tianjin 300350, P. R. China；

[2]Tianjin Key Laboratory of Pulp and Paper, Tianjin University of Science and Technology, Tianjin 300457, P. R. China；

[3]Department of Chemistry, College of Science, King Saud University, Riyadh 11451, Saudi Arabia.

[4]Department of Chemistry, University of North Texas 1508 W Mulberry St, Denton, TX 76201 United States.

E-mail: libaiyan@nankai.edu.cn; yangbingwen@tust.edu.cn; Shengqian.Ma@unt.edu

Table of Contents

Section 1. Supplementary Methods S3

Section 2. Supplementary Notes S9

Section 3. Supplementary References S51

Section 1. Supplementary Methods

1.1 Synthetic Procedures

Synthesis of 1, 3, 5-Triformylphloroglucinol

1, 3, 5-Triformylphloroglucinol (Tp) was synthesized according to the reported procedure with some minor modification[1]. Dried phloroglucinol (99%, 12.0 g, 98 mmol) and trifluoroacetic acid (99.5%, 240 mL) was added into a 1 L three-necked round-bottom flask and stirred for 5 min at 50 °C, then hexamethylenetetramine (99.5%, 42 g, 300 mmol) was added into the mixture slowly and refluxed at 120 °C under N_2 for 3 h. Then 3 M HCl (36～38%, 300 mL) was added slowly, the mixture was heated at 100 °C for 1 h. After cooling to room temperature, the solution was filtered through celite, extracted with CH_2Cl_2 (99.9%) for five times, dried over $MgSO_4$ (99.5%), and the solution was evaporated under reduced pressure to afford light yellow solid. Yield: 6.4 g (30.3%). ^1H NMR (300 MHz, $CDCl_3$) δ 14.10 (s, 3H, OH), 10.14 (s, 3H, CHO) ppm.

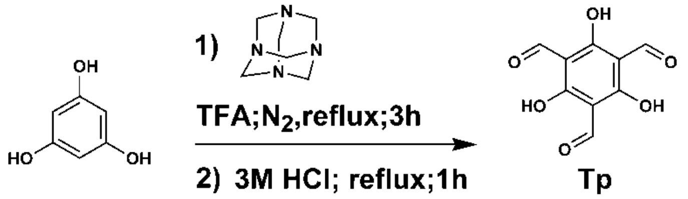

Synthesis of C4RACHO

C4RACHO was synthesized by a published procedure with some minor modification[2]. Resorcin[4]arene (98%, 24.24 g, 32 mmol) and hexamethylenetetramine (99.5%, 32 g, 228 mmol) were put in a 350 mL thick-walled pressure bottle. Trifluoroacetic acid (99.5%, 200 mL) was added and stirred vigorously until the substrates were dispersed in the liquid. The suspension was heated to 120 °C with stirring for 1 h. The resulting dark solution was poured into a flask containing $CHCl_3$ (99.5%, 500 mL) and aqueous HCl (36～38%, 500 mL, 1 M). The mixture was stirred vigorously overnight. The organic phase was separated, and the aqueous phase was washed with $CHCl_3$ (99.5%, 500 mL) several times. The combined chloroform extracts were dried over anhydrous

$MgSO_4$ (99.5%) and evaporated to dryness. The resulting crude precipitate was washed with acetone (99.5%, 500 mL), filtered off, and vacuum-dried to afford 14.72 g of the yellow product (56%). ^1H NMR ($CDCl_3$/500 MHz): δ 13.14 (bs, 4H), 10.30 (bs, 4H), 8.35 (bs, 4H), 7.38 (bs, 4H), 4.48 (bt, 4H), 2.05 (bq, 8H), 1.46 (bm, 4H), 1.01 (bd, 24H).

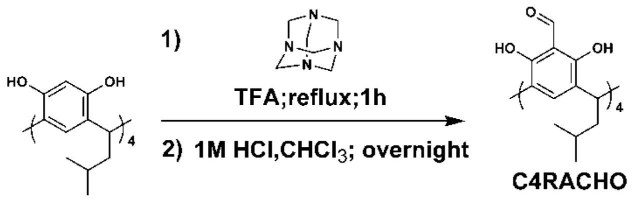

Synthetic procedures for HPH-ZIF-8

General method for the synthesis of HPH-ZIF-8 in water via high pressure homogenization (Laboratory synthesis, discharge: 20 L h^{-1}). The synthesis solution with molar ratio of Zn^{2+}: 2-methylimidazole:H_2O =1:40:706 was prepared as follows. Firstly, $Zn(NO_3)_2{\cdot}6H_2O$ (99%, 2.34 g, 0.00786 mol) was dissolved in 10.0 mL water. Secondly, 2-Methylimidazole (Hmim, 98%, 25.8 g, 0.314 mol) was dissolved in 90 mL water. The zinc nitrate solution was then mixed with the 2-methylimidazole solution under stirring. The mixture was pumped into homogenizer and homogenized under 100 MPa for 1, 2, 10, 30 min, respectively. After this time interval, the white MOF powders were collected by filtration and the 2-methylimidazole solution is recycled. The obtained powders were washed with water for 3 times and finally dried under vacuum at 100 °C for 12 hours.

Synthetic procedures for HPH-ZIF-67

General method for the synthesis of HPH-ZIF-67 in water via high pressure homogenization (Laboratory synthesis, discharge: 20 L h^{-1}). 2-Methylimidazole (Hmim, 98%, 55.0 g, 0.670 mol), $CoCl_2{\cdot}6H_2O$ (99.5%, 4.5 g, 0.019 mol) was dissolved in MeOH (98%, 130 mL). Mixture was pumped into homogenizer and homogenized in 100 MPa for 1, 2, 10, 30 min, respectively. After this time interval, the purple MOF powders were collected by filtration and the 2-methylimidazole

solution is recycled. The powders were washed with MeOH (98%) for 3 times and dried under vacuum at 100 °C for 12 hours.

Synthetic procedures for HPH-NH_2-MIL-53(Al)

General method for the synthesis of NH_2-MIL-53(Al) in water via high pressure homogenization (Laboratory synthesis, discharge: 20 L h^{-1}). H_2O (300 mL), 2-aminoterephthalic acid (NH_2-H_2BDC, ⩾98%, 37.5 g, 0.207 mol), and NaOH (analytical pure, 17.9 g, 0.45 mol) was added into a 500 mL beaker to obtain a solution 1. H_2O (250 mL), and $AlCl_3·6H_2O$ (98%, 50 g, 0.207 mol) was added into a 500 mL beaker to obtain a solution 2. Solution 2 was slowly added to solution 1 under stirring, leading to the immediate appearance of a yellow solid, which became a gel-like mixture along the reaction time. Mixture was pumped into homogenizer and homogenized under 100 MPa for 1, 2, 10, 30 min, respectively. After this time interval, the yellow brown MOF powders were collected and recovered by filtration, and it was washed with water for 3 times. Product was finally dried under vacuum at 120 °C for 12 hours.

Synthetic procedures for HPH-CC3R-OH

General method for the synthesis of CC3R-OH via high pressure homogenization (Laboratory synthesis, discharge: 20 L h^{-1}). 2-hydroxy-1,3,5-triformylbenzene (98%, 1068.8 mg, 6 mmol), and (1R,2R)-1,2-diaminocyclohexane (99.9%, 1027.8 mg，9 mmol), and methanol (98%, 60 mL) was added into a 500 mL beaker and stirred for 5 min. The mixture was pumped into homogenizer and homogenized under 100 MPa for 2, 5, 10, 30 min, respectively. After this time interval, the yellow POC powders were collected by filtration, and it was washed with methanol (98%) for 3 times. The product was finally dried under vacuum at 150 °C for 12 hours.

Synthetic procedures of COFs using solvothermal method

TpPa-1 was synthesized according to the literature[3], typically, Tp (98%, 63 mg, 0.3 mmol), and p-phenylenediamine (Pa-1, 99%, 48 mg, 0.45 mmol) was weighed into a Pyrex tube (o.d. × i.d. = 10 × 8 mm^2 and length 18 cm), and the mixture was added

into mesitylene (99%, 1.5 mL), dioxane (99%, 1.5 mL), and 3 M aqueous acetic acid (99.5%, 0.5 mL). The obtained suspension was sonicated for 10 minutes. The tube was flash frozen in a liquid nitrogen bath and degassed by three freeze-pump-thaw cycles. The tube was sealed off and then placed in an oven at 120 °C for 3 days. The resulting precipitate was filtered and washed with anhydrous acetone (99.5%). The obtained powder was solvent exchanged with anhydrous acetone (99.5%) 5-6 times and then dried at 180 ℃ under vacuum for 24 hours. IR (powder, cm^{-1}): 1582 (C=C), 1257 (C-N).

TpPa-2 was synthesized according to the literature[3], typically, Tp (98%, 63 mg, 0.3 mmol), and 2,5-dimethyl-p-phenylenediamine (Pa-2, 98%, 61 mg, 0.45 mmol) was weighed into a Pyrex tube (o.d. × i.d. = 10 × 8 mm^2 and length 18 cm), and the mixture was added into mesitylene (99%, 1.5 mL), dioxane (99%, 1.5 mL), 3 M aqueous acetic acid (99.5%, 0.5 mL). The obtained suspension was sonicated for 10 minutes. The tube was flash frozen in a liquid nitrogen bath and degassed by three freeze-pump-thaw cycles. The tube was sealed off and then placed in an oven at 120 °C for 3 days. The resulting precipitate was filtered and washed with anhydrous acetone (99.5%). The obtained powder was solvent exchanged with anhydrous acetone (99.5%) 5-6 times and then dried at 180 ℃ under vacuum for 24 hours. IR (powder, cm^{-1}): 1582 (C=C), 1250 (C-N).

TpBD was synthesized according to the literature[4], typically, Tp (98%, 63 mg, 0.3 mmol), and Benzidine (BD, 98%, 82.9 mg, 0.45 mmol) was weighed into a Pyrex tube (o.d. × i.d. = 10 × 8 mm^2 and length 18 cm), and the mixture was added into mesitylene (99%, 1.5 mL), dioxane (99%, 1.5 mL), 3 M aqueous acetic acid (99.5%, 1.5 mL). The obtained suspension was sonicated for 10 minutes. The tube was flash frozen in a liquid nitrogen bath and degassed by three freeze-pump-thaw cycles. The tube was sealed off and then placed in an oven at 120 °C for 3 days. The resulting precipitate was filtered and washed with anhydrous acetone (99.5%). The obtained powder was solvent exchanged with anhydrous acetone (99.5%) 5-6 times and then dried at 180 ℃ under vacuum for 24 hours. IR (powder, cm^{-1}): 1594 (C=C), 1258

(C-N).

DAAQ was synthesized according to the literature[4], typically, Tp (98%, 20 mg, 0.096 mmol), and 2,6-diaminoanthraquinone (AQ, 98%, 34 mg, 0.142 mmol) was weighed into a glass ampoule, and the mixture was added into dioxane (99%, 1 mL), and the resulting suspension was sonicated briefly (20 s). A 50 μL of 6 M acetic acid (99.5%) was added. The ampoule was frozen, placed under partial vacuum (100 mTorr) and flame sealed. The ampoule was then placed in an oven at 120 °C for 3 days. The resulting precipitate was filtered and placed in a 50 mL fritted funnel and soaked in N,N-Dimethylformamide (DMF, 99.8%) until the filtrate was clear. Then, the powder was washed with water and solvent exchanged with anhydrous acetone (99.5%) 2 times and then dried at 90 °C under dynamic vacuum for 12 h. IR (powder, cm^{-1}): 1615 (C=O), 1250 (C-N).

Large-scale synthetic procedures for HPH-TpPa-1

1,3,5-triformylphloroglucinol (Tp, 98%, 37.8 g, 180 mmol) and p-phenylenediamine (Pa-1, 99%, 28.8 g, 270 mmol) were put into water-acetic acid medium (water, 6 L, acetic acid, 99.5%, 3 L). The mixture is stirred for 5 min to achieve a homogenous suspension and then the mixture was pumped into homogenizer (large-scale synthesis, discharge: 180 L h^{-1}) and homogenized for 30 min in 100 MPa. The COF powders were collected and filtered, washed with water and ethanol (99.5%) for 2-3 times and finally dried under vacuum at 150 °C for 12 hours (yield, 54.6 g, 96%).

Large-scale synthetic procedures for HPH-HKUST-1

Trimesic acid (99%, 121.7 g, 0.58 mol) was dissolved in ethanol (99.5%, 1.8 L). Afterwards, a suspension of $Cu(OH)_2$ (99 %, 90.7 g, 0.93 mol) in water (0.7 L) was added to achieve a mixture. The mixture was pumped into homogenizer (large-scale synthesis, discharge: 180 L h^{-1}) and homogenized under 100 MPa for 2 min. The blue MOF powders were collected and filtered, washed with water and ethanol (99.5%) for 3 times and finally dried under vacuum at 120 °C for 12 hours to obtain the final product (yield, 188.9 g, 99%).

Large-scale synthetic procedures for HPH-CPOC-301

C4RACHO (49.2 g, 99%, 60 mmol), p-Phenylenediamine (99%, 12.96 g, 30 mmol), and mesitylene (99%, 1.5 L) was added into a beaker and stir for 5 min. The mixture was pumped into homogenizer (large-scale synthesis, discharge: 180 L h^{-1}) and homogenized under 100 MPa for 2 min. The suspension was allowed standing and the powder was collected by filtration, and it was washed with ethyl ether (99.5%) for 3 times. The product was finally dried under vacuum at 150 °C for 12 hours (yield, 53.2 g, 92%).

Kilogram-scale production of HPH-HKUST-1

To illustrate the feasibility of HPH approach for kilogram-scale production of crystalline porous materials, we selected HKUST-1 as proof of concept. Typically, trimesic acid (99%, 654 g, 3.12 mol) was dissolved in ethanol (99.5%, 9 L). Afterwards, a suspension of $Cu(OH)_2$ (99%, 487 g, 5 mol) in water (3 L) was added to achieve a mixture. The mixture was pumped into homogenizer (large-scale synthesis, discharge: 180 L h^{-1}) and homogenized under 100 MPa for 12 min. Let the suspension stand for 1 h, about 6 L settled solution was dumped away. The remaining suspension is removed by centrifugation (total centrifugal volume of 3 L for each operation). The blue MOF powders were washed with water and ethanol (99.5%) for 3 times and finally dried under vacuum at 120 °C for 12 hours to obtain the final product (Supplementary Fig. 73, yield, 1015 g, 99%).

1.2 Calculation of STY

Space–time yield (STY) is an important parameter for the industrial production of the product[6] and can be determined by equation. 1:

$$\mathrm{STY} = \frac{\mathrm{m}}{\mathrm{V_{solution}} \cdot \tau} \times 1.44 \times 10^{6} \qquad (1)$$

where m represents the pure powder mass (g) of the HPH-COFs, HPH-MOFs, and

HPH-POCs, respectively. $V_{solution}$ represents the total volume (cm^3) of the mixture, and τ represents the reaction time (min) where C_0 (mg/L) and C_t (mg/L) are the initial and residual concentration of pollutant in the stock solution and filtrate, respectively.

Section 2. Supplementary Notes

2.1 Supplementary Figures

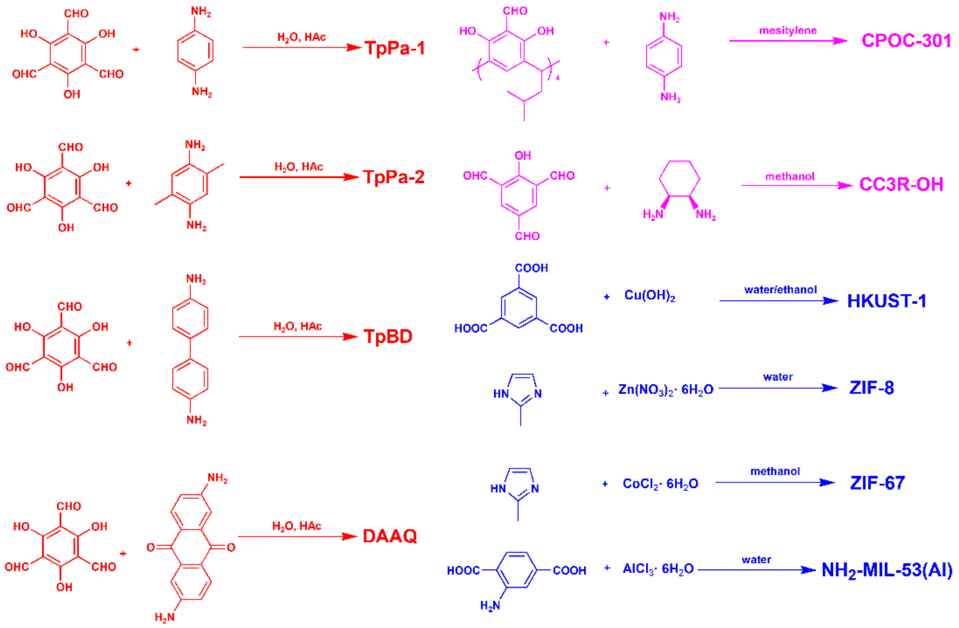

Supplementary Fig. 1. General procedure for synthesizing crystalline porous materials including COFs, MOFs, and POCs via high pressure homogenization (HPH) approach.

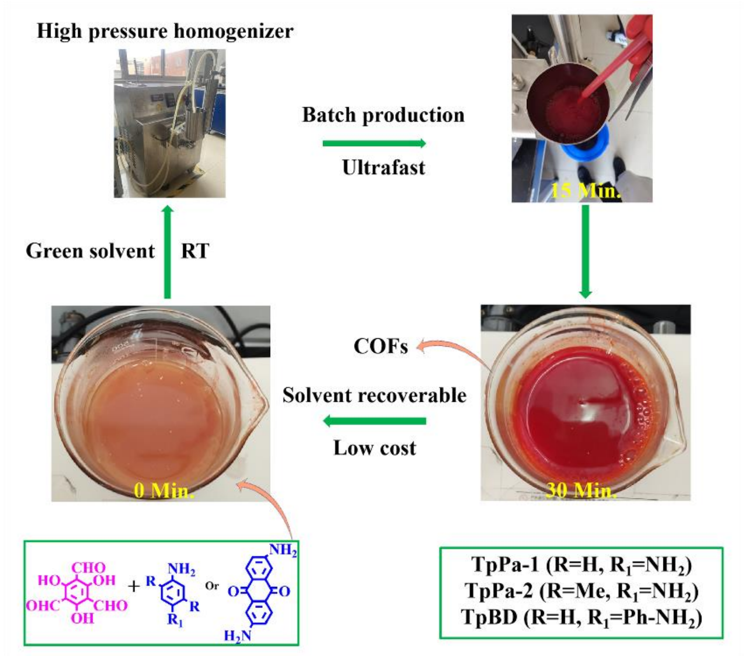

Supplementary Fig. 2. Schematic representation of HPH approach for synthesizing HPH-TpPa-1, HPH-TpPa-2, HPH-TpBD, and HPH-DAAQ.

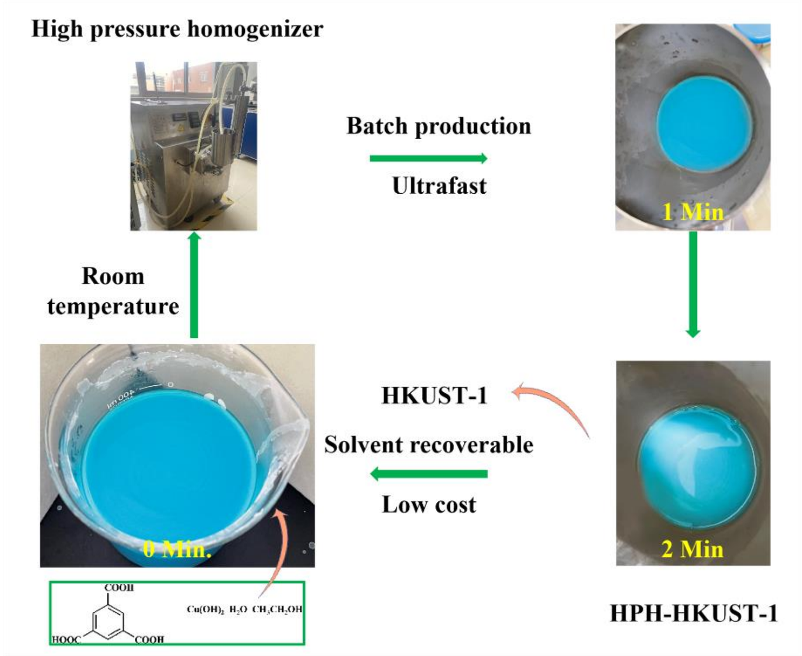

Supplementary Fig. 3. Schematic representation of HPH approach for synthesizing HPH-HKUST-1.

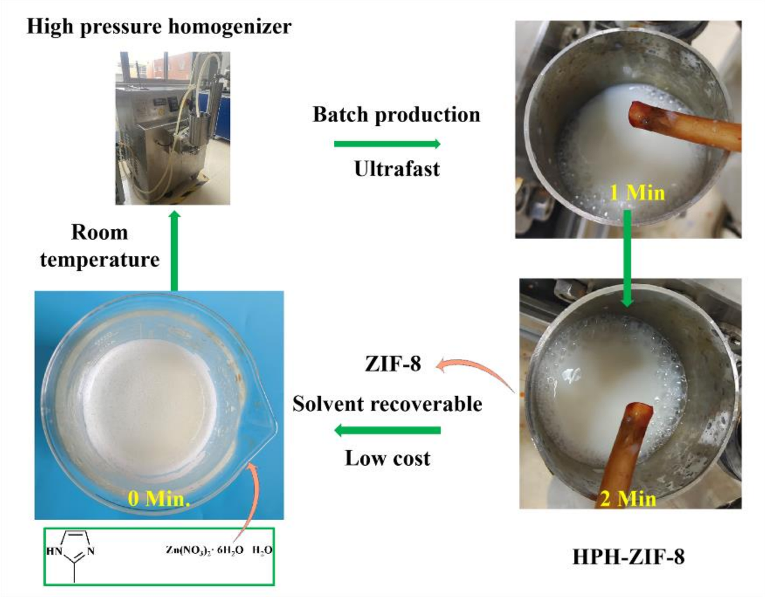

Supplementary Fig. 4. Schematic representation of HPH approach for synthesizing HPH-ZIF-8.

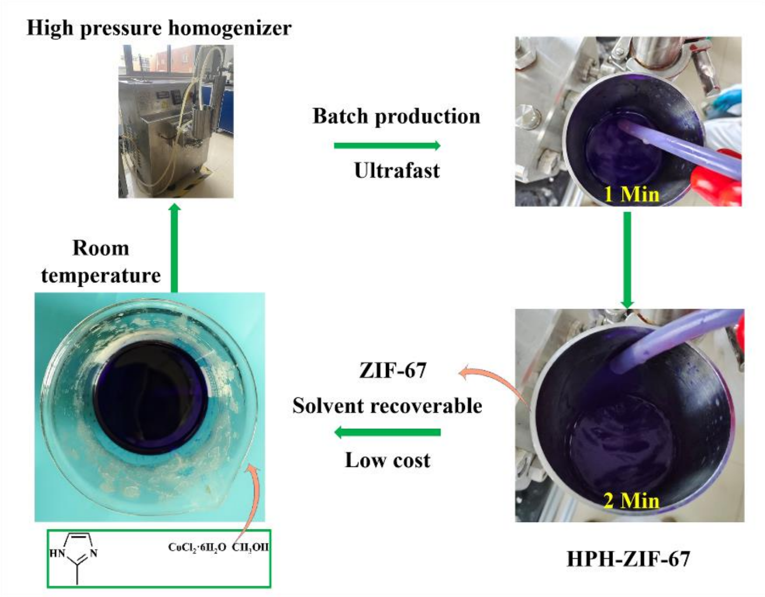

Supplementary Fig. 5. Schematic representation of HPH approach for synthesizing HPH-ZIF-67.

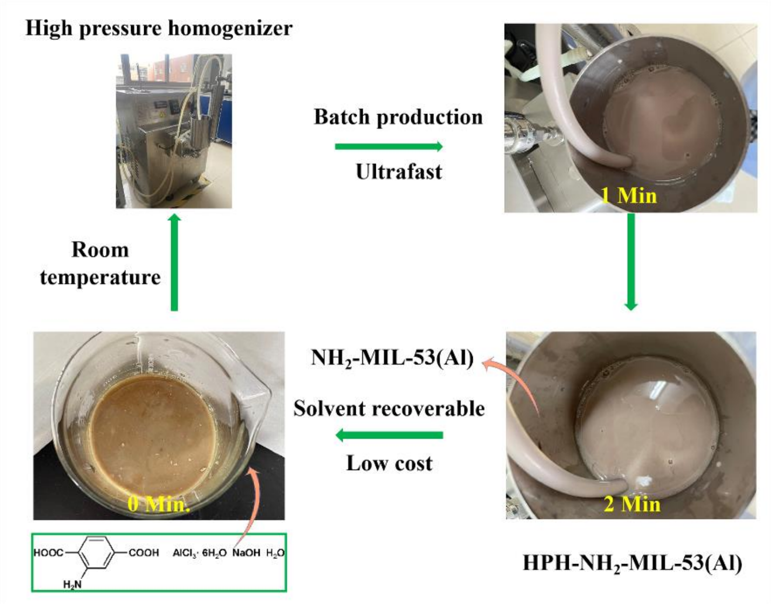

Supplementary Fig. 6. Schematic representation of HPH approach for synthesizing HPH-NH_2-MIL-53(Al).

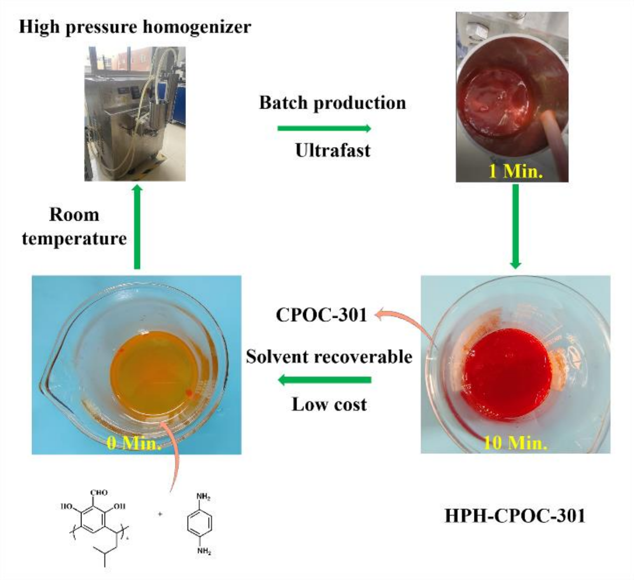

Supplementary Fig. 7. Schematic representation of HPH approach for synthesizing HPH-CPOC-301.

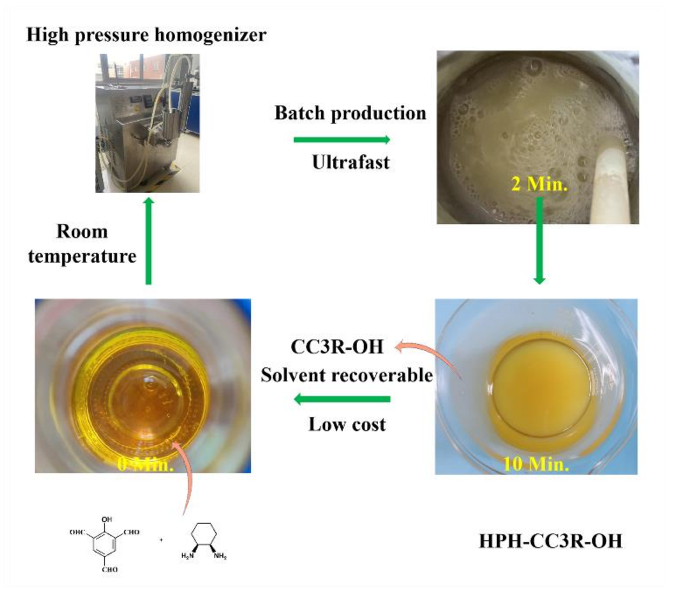

Supplementary Fig. 8. Schematic representation of HPH approach for synthesizing HPH-CC3R-OH.

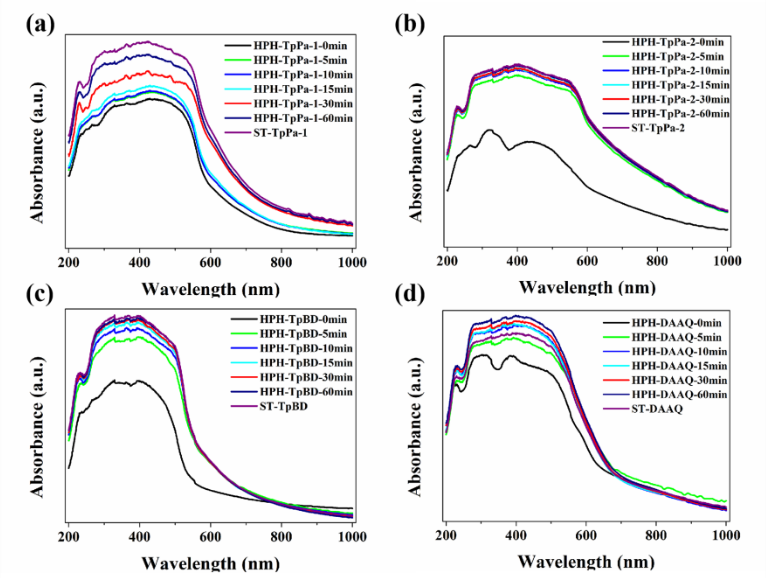

Supplementary Fig. 9. The comparison of UV–vis spectra of HPH-COFs formed at different time, and ST-COFs. (a) HPH-TpPa-1 formed at different time, and ST-TpPa-1, (b) HPH-TpPa-2 formed at different time, and ST-TpPa-2, (c) HPH-TpBD formed at different time, and ST-TpBD, (d) HPH-DAAQ formed at different time, and ST- DAAQ.

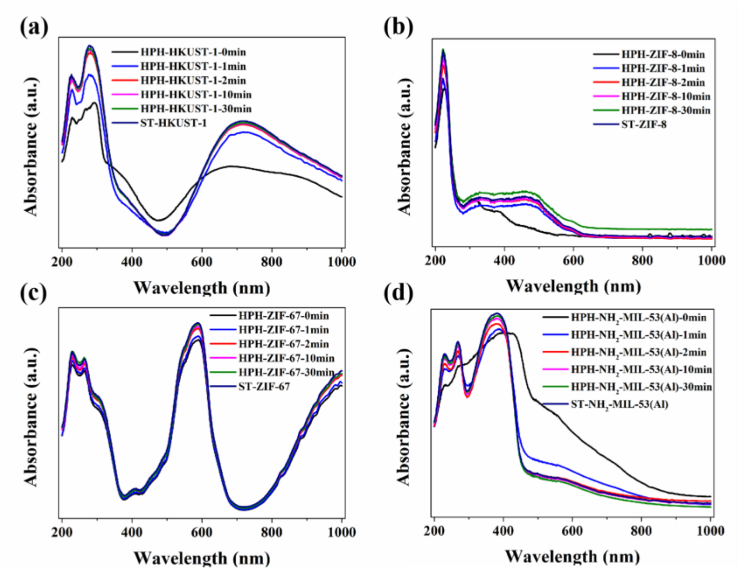

Supplementary Fig. 10. The comparison of UV–vis spectra of HPH-MOFs formed at different time, and ST-MOFs. (a) HPH-HKUST-1 formed at different time, and ST-HKUST-1, (b) HPH-ZIF-8 formed at different time, and ST-ZIF-8, (c) HPH-ZIF-67 formed at different time, and ST-ZIF-67, (d) HPH-NH_2-MIL-53(Al) formed at different time, and ST-NH_2-MIL-53(Al).

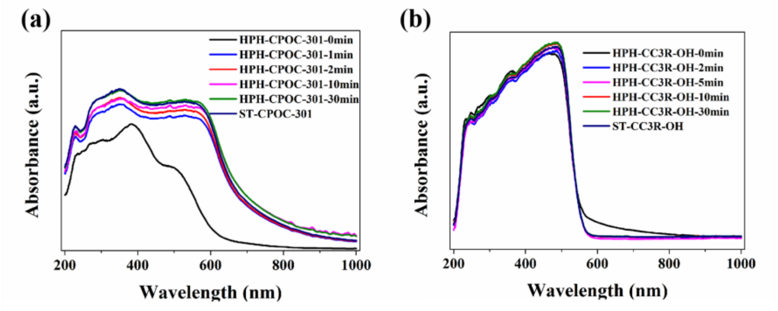

Supplementary Fig. 11. The comparison of UV–vis spectra of HPH-POCs formed at different time, and ST-POCs. (a) HPH-CPOC-301 formed at different time, and ST-CPOC-301, (b) HPH-CC3R-OH formed at different time, and ST-CC3R-OH.

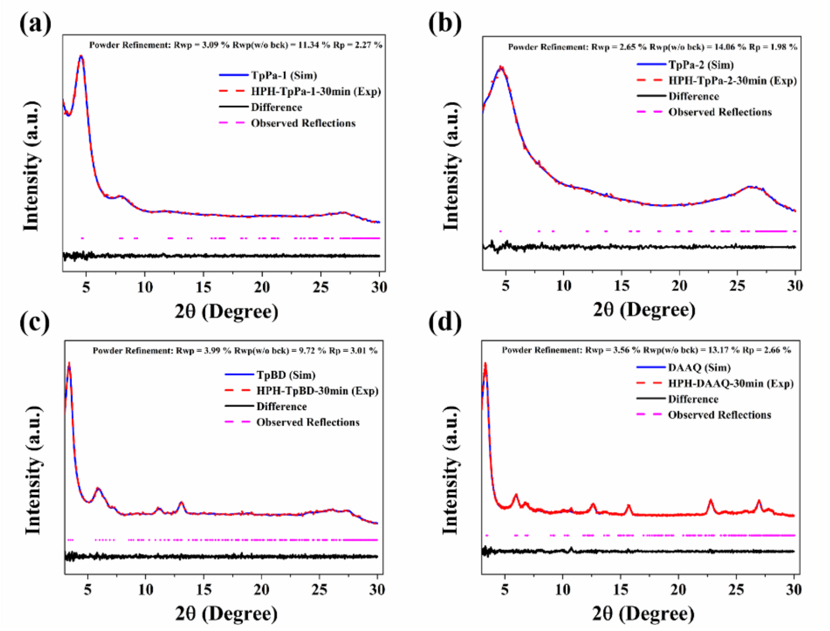

Supplementary Fig. 12. The comparison of PXRD patterns of the as-synthesized COFs via HPH approach and their Pawley refinements. (a) HPH-TpPa-1-30min, (b) HPH-TpPa-2-30min, (c) HPH-TpBD-30min, (d) HPH-DAAQ-30min. Note, all the abbreviation of a.u. in the PXRD patterns of this manuscript represent for arb.units.

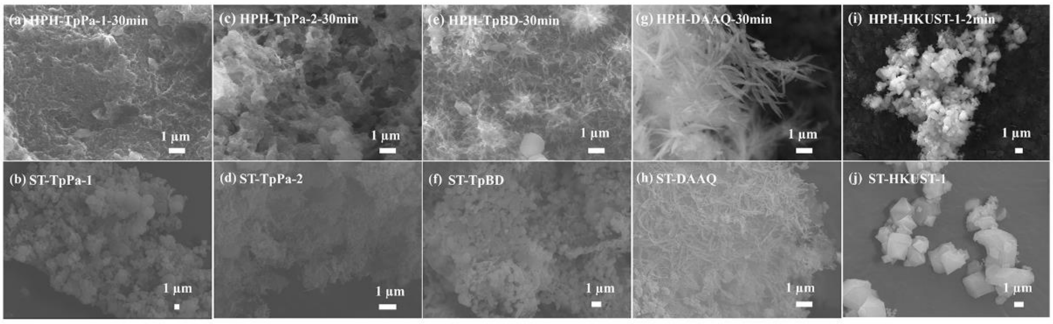

Supplementary Fig. 13. The SEM images of (a) HPH-TpPa-1-30min, (b) ST-TpPa-1 (72 h), (c) HPH-TpPa-2-30min, (d) ST-TpPa-2 (72 h), (e) HPH-TpBD-30min, (f) ST-TpBD (72 h), (g) HPH-DAAQ-30min, (h) ST-DAAQ (48 h), (i) HPH-HKUST-1-2min, (j) ST-HKUST-1 (8 h).

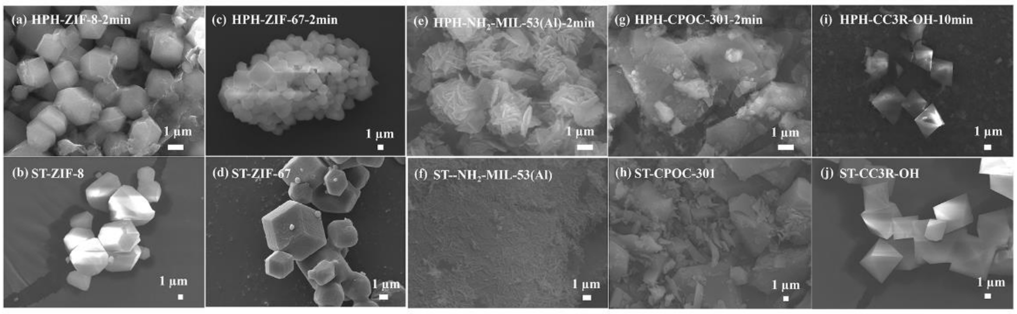

Supplementary Fig. 14. The SEM images of (a) HPH-ZIF-8-2min, (b) ST-ZIF-8 (48 h), (c) HPH-ZIF-67-2min, (d) ST-ZIF-67 (72 h), (e) HPH-NH_2-MIL-53(Al)-2min, (f) ST-NH_2-MIL-53(Al) (72 h), (g) HPH-CPOC-301-2min, (h) ST-CPOC-301 (48 h, 1 week under room temperature), (i) HPH-CC3R-OH-10min, (j) ST-CC3R-OH (4 h).

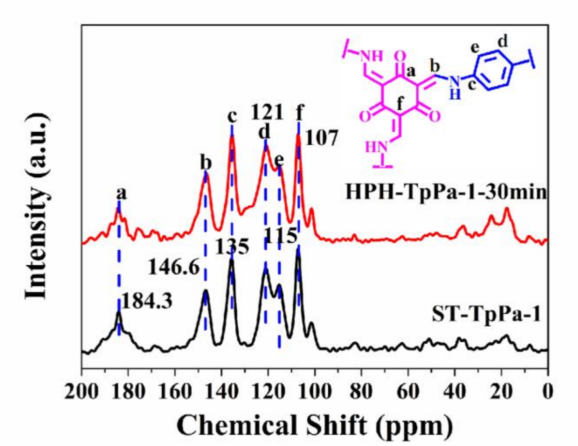

Supplementary Fig. 15. The comparison of solid state ^{13}C NMR of TpPa-1 based on HPH (red) and ST (black) methods.

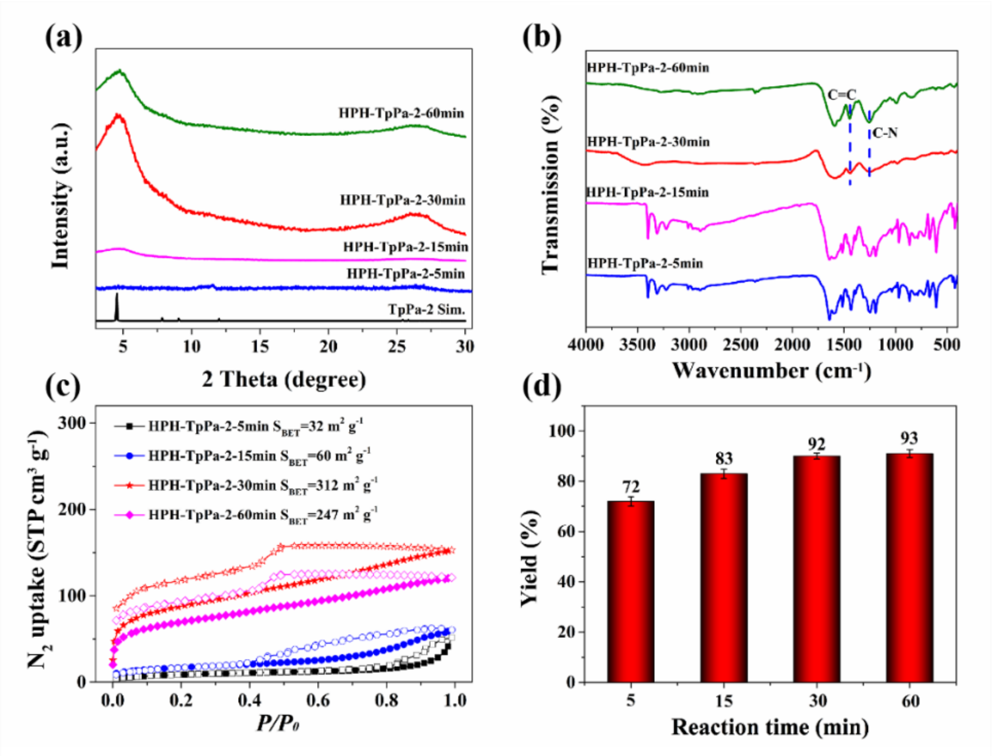

Supplementary Fig. 16. (a) PXRD patterns of HPH-TpPa-2 at different reaction times. (b) FT-IR spectra of HPH-TpPa-2 at different reaction times, the peaks located at ~1582 cm^{-1} and 1250 cm^{-1} correspond to the C=C, and C–N[7]. (c) N_2 sorption isotherms of HPH-TpPa-2 at different reaction times. (d) The yields of HPH-TpPa-2 at different reaction times. All the error bars in this figure represent the standard deviation (n = 3 independent experiments), data are presented as mean values ± SD.

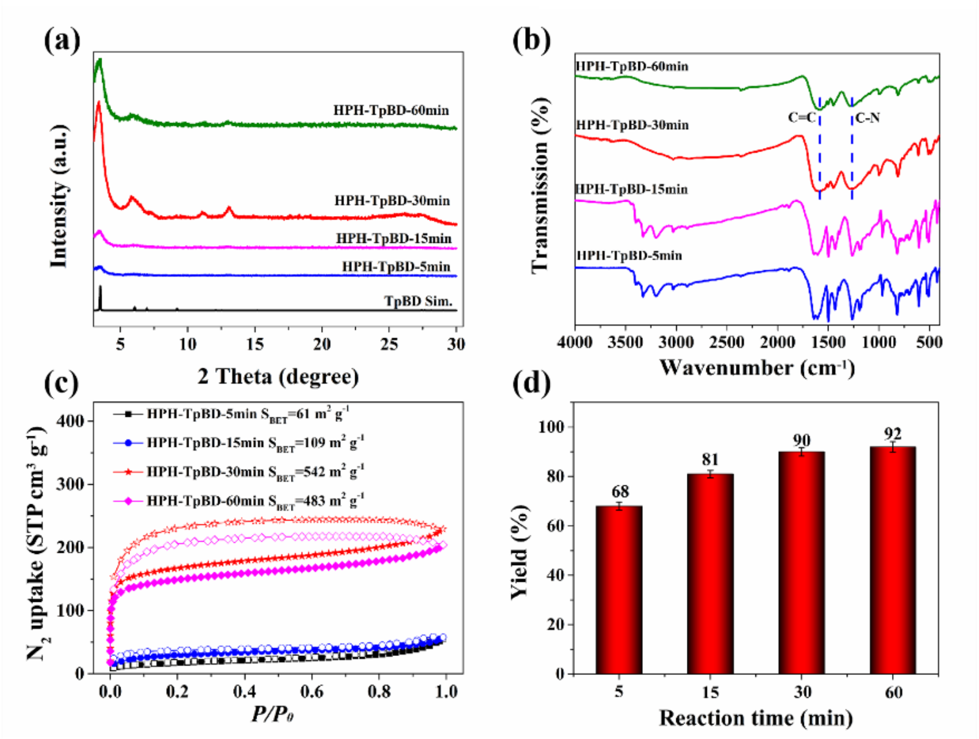

Supplementary Fig. 17. (a) PXRD patterns of HPH-TpBD at different reaction times. (b) FT-IR spectra of HPH-TpBD at different reaction times, several peaks located at ~1578 cm^{-1} and 1267 cm^{-1} can be attributed to the C=C, and C–N[8]. (c) N_2 sorption isotherms of HPH-TpBD at different reaction times. (d) The yields of HPH-TpBD at different reaction times. All the error bars in this figure represent the standard deviation (n = 3 independent experiments), data are presented as mean values ± SD.

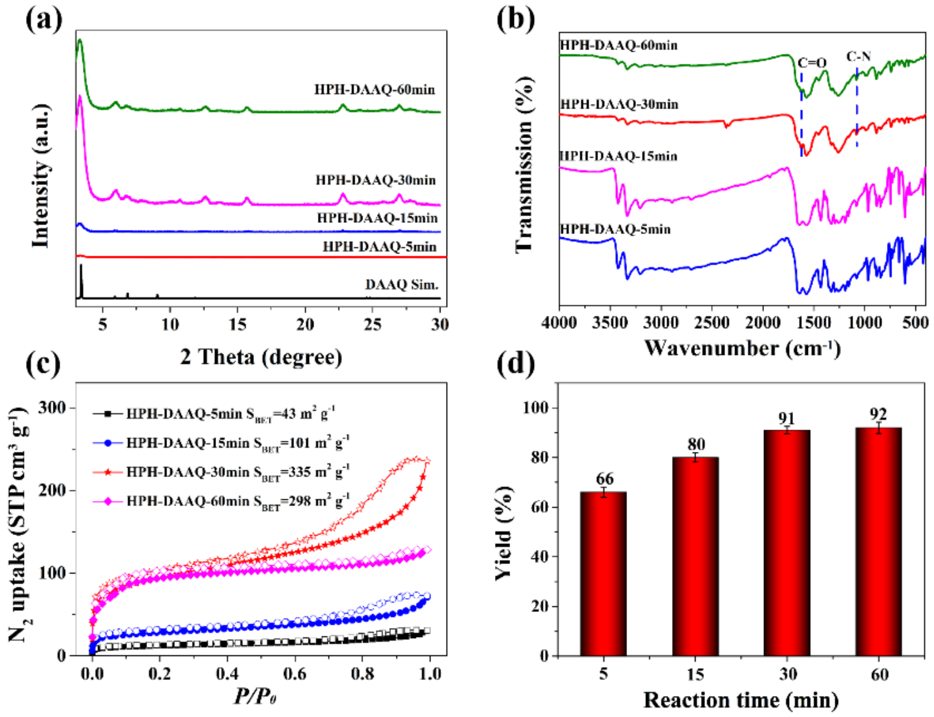

Supplementary Fig. 18. (a) PXRD patterns of HPH-DAAQ at different reaction times. (b) FT-IR spectra of HPH-DAAQ at different reaction times, several peaks located at ~1615 cm^{-1} and 1250 cm^{-1} can be attributed to the C=O, and C–N[8]. (c) N_2 sorption isotherms of HPH-DAAQ at different reaction times. (d) The yields of HPH-DAAQ at different reaction times. All the error bars in this figure represent the standard deviation (n = 3 independent experiments), data are presented as mean values ± SD.

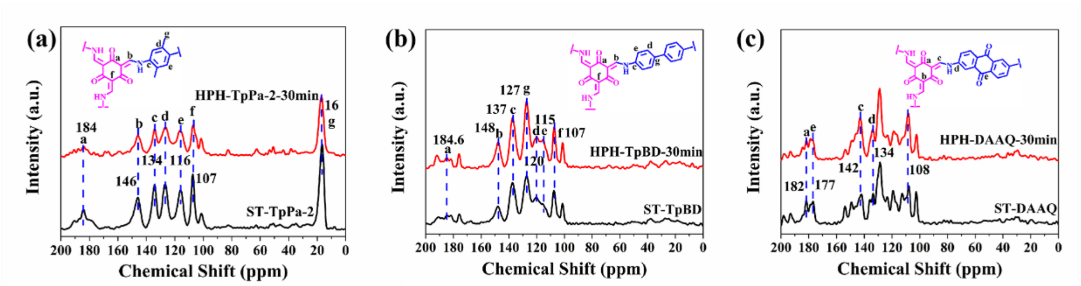

Supplementary Fig. 19. The comparison of solid state ^{13}C NMR of (a) TpPa-2, (b) TpBD, (c) DAAQ based on HPH (red) and ST (black) methods.

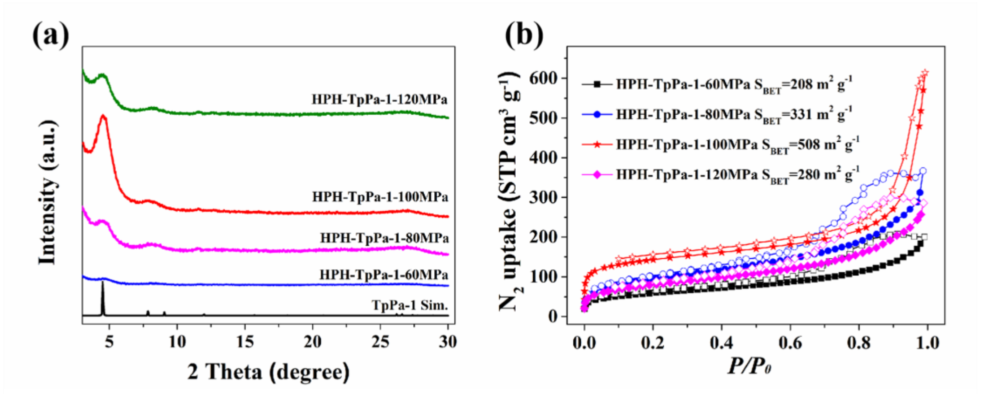

Supplementary Fig. 20. (a) PXRD patterns of HPH-TpPa-1 obtained by high pressure homogenization under different homogenization pressures for 30 min. (b) N_2 sorption isotherms of HPH-TpPa-1 obtained by high pressure homogenization under different homogenization pressures for 30 min.

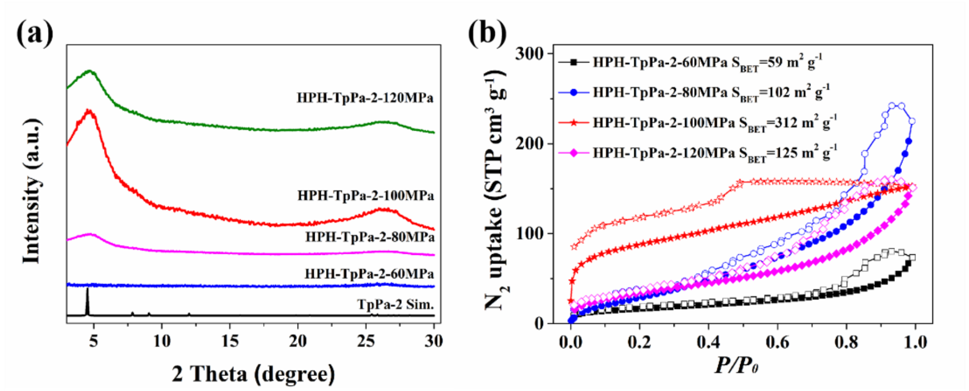

Supplementary Fig. 21. (a) PXRD patterns of HPH-TpPa-2 obtained by high pressure homogenization under different homogenization pressures for 30 min. (b) N_2 sorption isotherms of HPH-TpPa-2 obtained by high pressure homogenization under different homogenization pressures for 30 min.

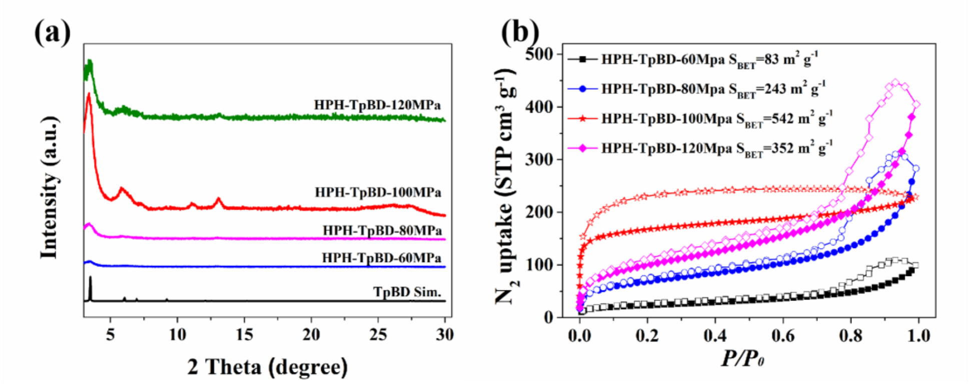

Supplementary Fig. 22. (a) PXRD patterns of HPH-TpBD obtained by high pressure homogenization under different homogenization pressures for 30 min. (b) N_2 sorption isotherms of HPH-TpBD obtained by high pressure homogenization under different homogenization pressures for 30 min.

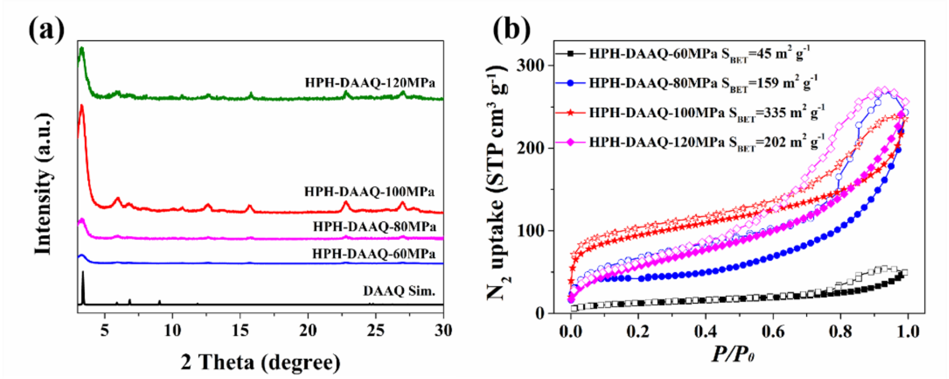

Supplementary Fig. 23. (a) PXRD patterns of HPH-DAAQ obtained by high pressure homogenization under different homogenization pressures for 30 min. (b) N_2 sorption isotherms of HPH-DAAQ obtained by high pressure homogenization under different homogenization pressures for 30 min.

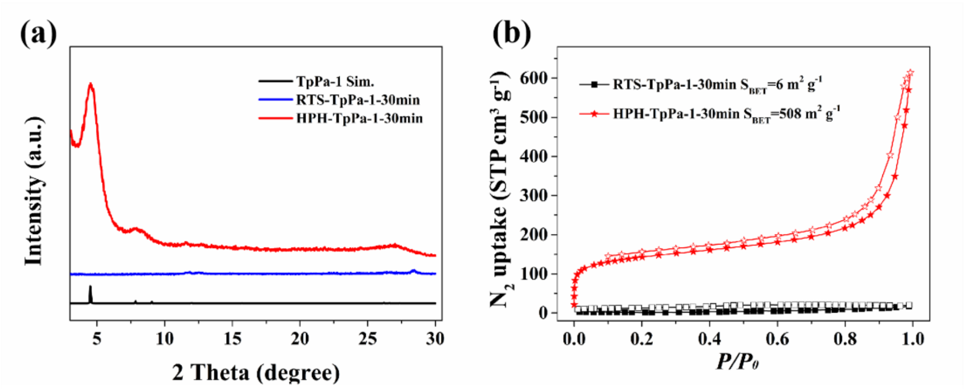

Supplementary Fig. 24. (a) PXRD patterns of HPH-TpPa-1 obtained by high pressure homogenization under 100 MPa for 30 min and TpPa-1 obtained via room temperature synthesis (RTS) with regular stir for 30 min. (b) N_2 sorption isotherms of HPH-TpPa-1 obtained by high pressure homogenization under 100 MPa for 30 min and TpPa-1 obtained via room temperature synthesis with regular stir for 30 min.

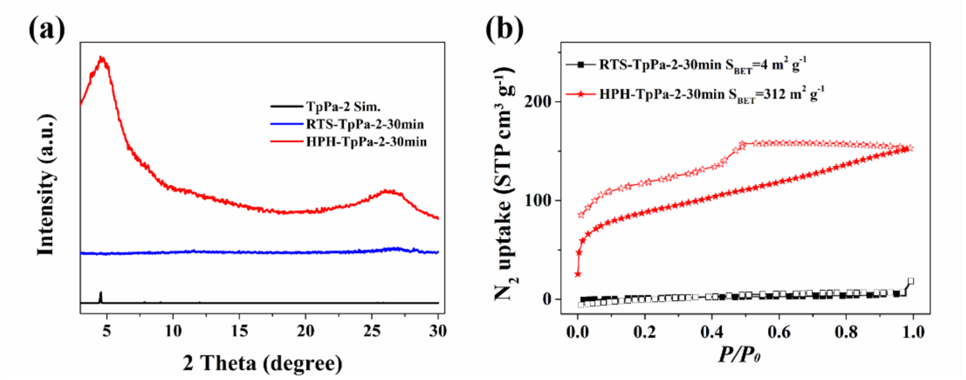

Supplementary Fig. 25. (a) PXRD patterns of HPH-TpPa-2 obtained by high pressure homogenization under 100 MPa for 30 min and TpPa-2 obtained via room temperature synthesis (RTS) with regular stir for 30 min. (b) N_2 sorption isotherms of HPH-TpPa-2 obtained by high pressure homogenization under 100 MPa for 30 min and TpPa-2 obtained via room temperature synthesis with regular stir for 30 min.

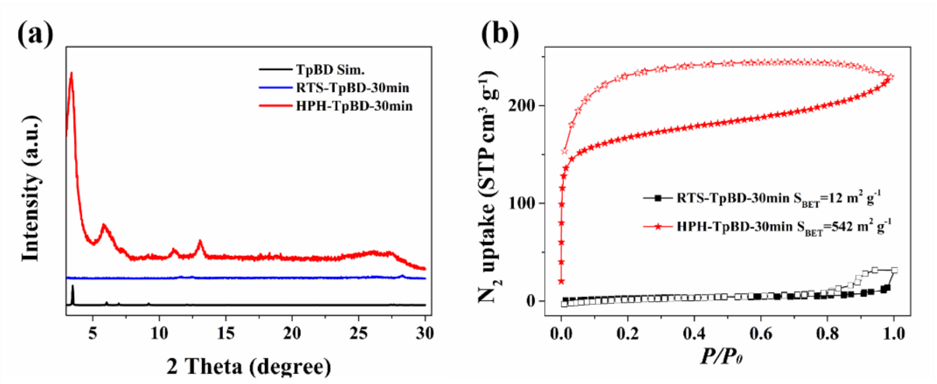

Supplementary Fig. 26. (a) PXRD patterns of HPH-TpBD obtained by high pressure homogenization under 100 MPa for 30 min and TpBD obtained via room temperature synthesis (RTS) with regular stir for 30 min. (b) N_2 sorption isotherms of HPH-TpBD obtained by high pressure homogenization under 100 MPa for 30 min and TpBD obtained via room temperature synthesis with regular stir for 30 min.

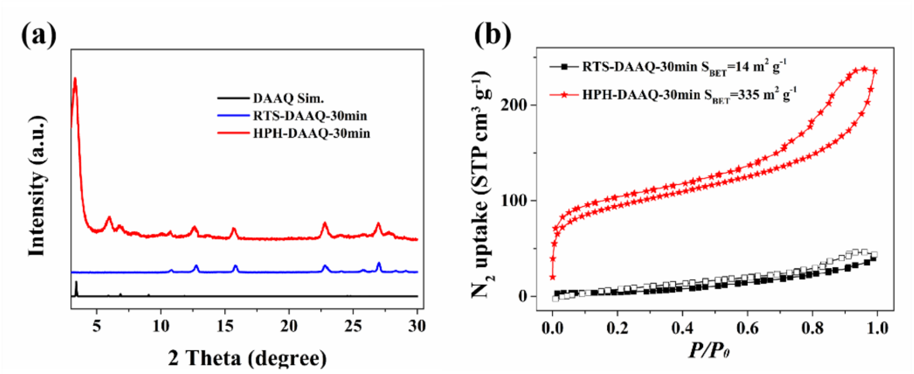

Supplementary Fig. 27. (a) PXRD patterns of HPH-DAAQ obtained by high pressure homogenization under 100 MPa for 30 min and DAAQ obtained via room temperature synthesis (RTS) with regular stir for 30 min. (b) N_2 sorption isotherms of HPH-DAAQ obtained by high pressure homogenization under 100 MPa for 30 min and DAAQ obtained via room temperature synthesis with regular stir for 30 min.

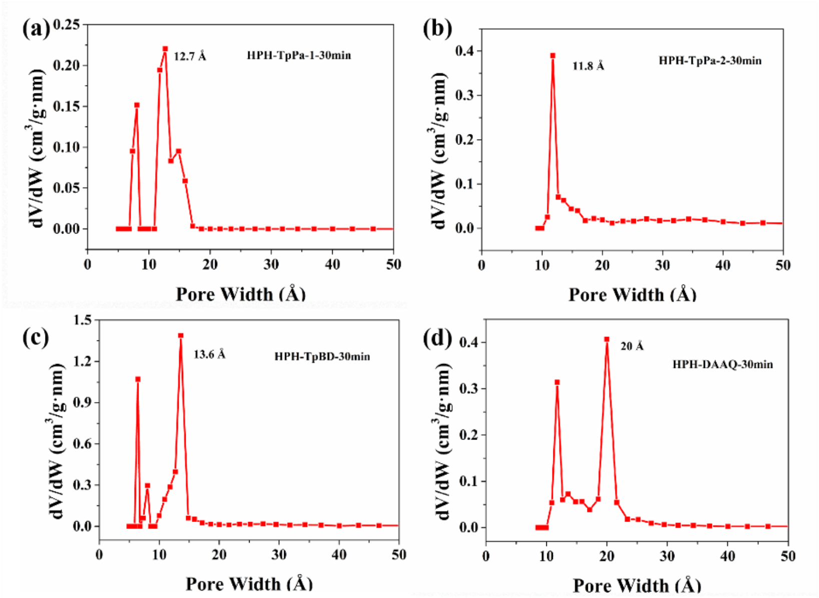

Supplementary Fig. 28. Pore size distribution of (a) HPH-TpPa-1-30min, (b) HPH-TpPa-2-30min, (c) HPH-TpBD-30min, (d) HPH-DAAQ-30min.

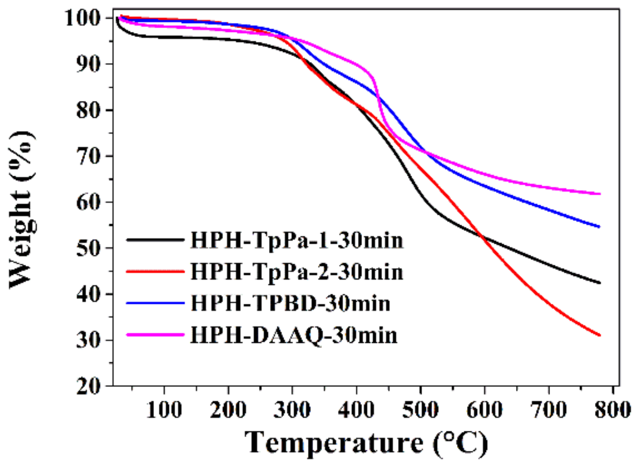

Supplementary Fig. 29. Thermogravimetric analysis of HPH-COFs.

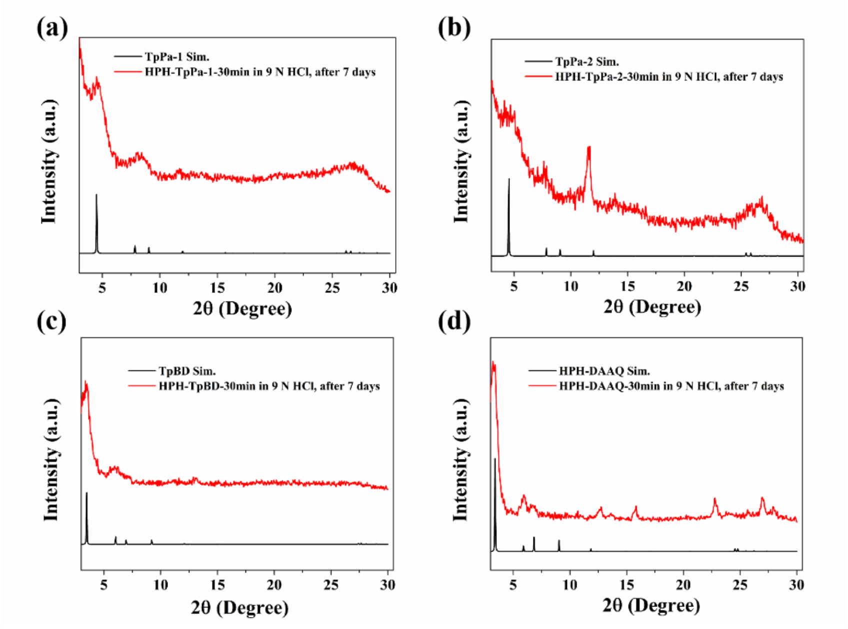

Supplementary Fig. 30. Acid stability test by dipping (a) HPH-TpPa-1-30min, (b) HPH-TpPa-2-30min, (c) HPH-TpBD-30min, (d) HPH-DAAQ-30min into 9 N HCl for 7 days.

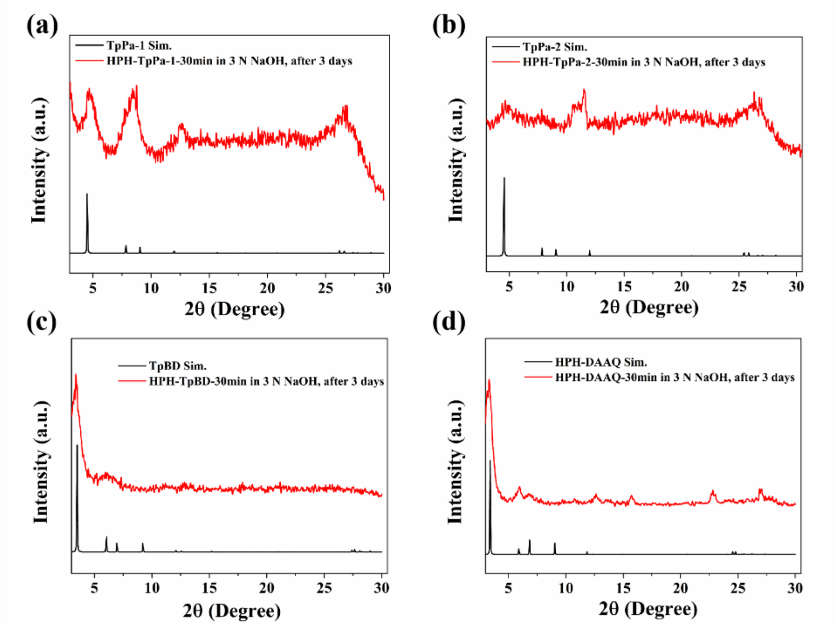

Supplementary Fig. 31. Base stability test by dipping (a) HPH-TpPa-1-30min, (b) HPH-TpPa-2-30min, (c) HPH-TpBD-30min, (d) HPH-DAAQ-30min into 3 N NaOH for 3 days.

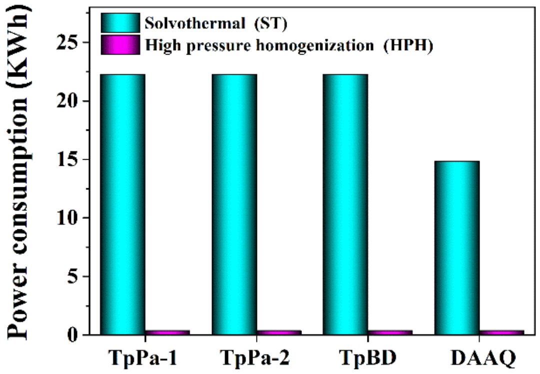

Supplementary Fig. 32. The comparison of power consumption of HPH-COFs and ST-COFs.

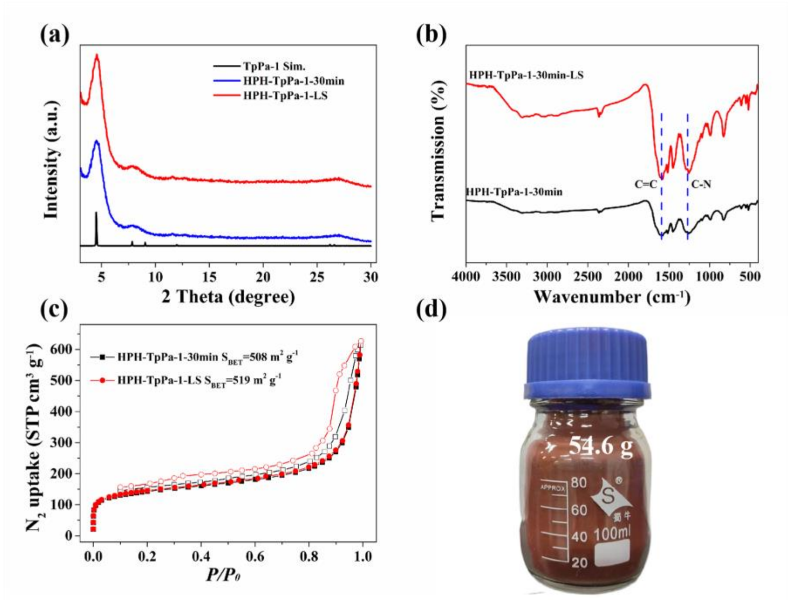

Supplementary Fig. 33. (a) The comparison of PXRD patterns of HPH-TpPa-1-30min and HPH-TpPa-1-LS (LS=Large-Scale). (b) The comparison of FT-IR spectra of HPH-TpPa-1-30min and HPH-TpPa-1-LS. (c) The comparison of N_2 sorption isotherms of HPH-TpPa-1-30min and HPH-TpPa-1-LS. (d) The photo of HPH-TpPa-1-LS obtained by high pressure homogenizer (discharge, 180 L h^{-1}).

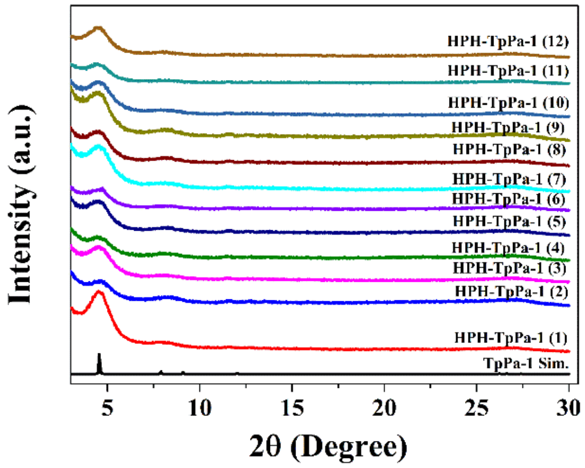

Supplementary Fig. 34. The PXRD of HPH-TpPa-1 obtained after solvent and catalyst reused. HPH approach can realize the readily recyclability of catalyst and solvent by adding 10 mL acetic acid after every six operations.

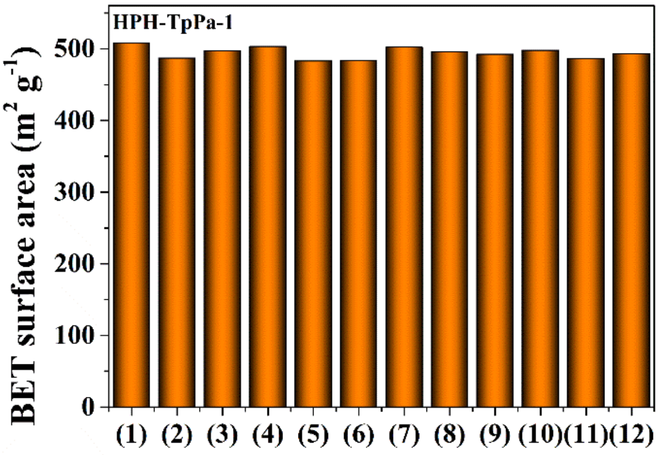

Supplementary Fig. 35. The BET surface area of HPH-TpPa-1 obtained after solvent and catalyst reused. HPH approach can realize the readily recyclability of catalyst and solvent by adding 10 mL acetic acid after every six operations.

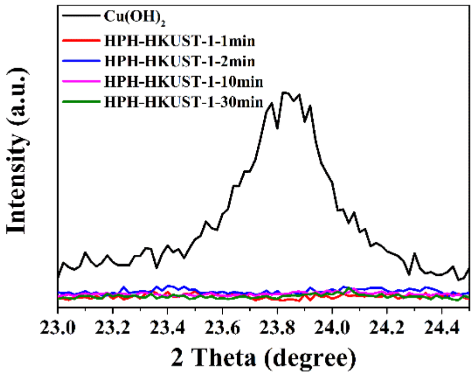

Supplementary Fig. 36. Crystallization evolution of HKUST-1 monitored by the disappear of PXRD peak in the range of 23–24.5° ($Cu(OH)_2$) for different time intervels.

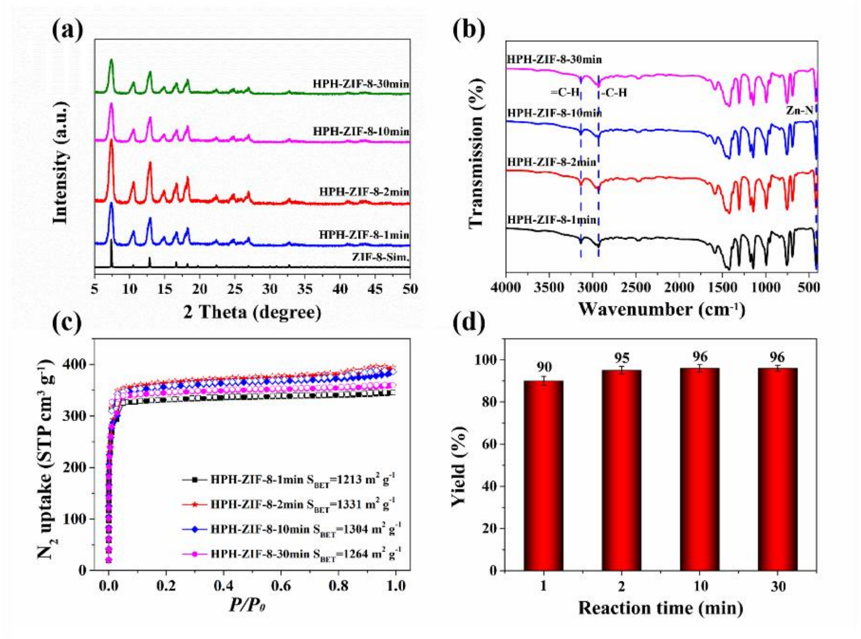

Supplementary Fig. 37. (a) PXRD patterns of HPH-ZIF-8 at different reaction times. (b) FT-IR spectra of HPH-ZIF-8 at different reaction times, several peaks located at ~3136, 2931 cm^{-1} and 421 cm^{-1} can be attributed to the =C-H, $–CH_3$ and Zn–N[9]. (c) N_2 sorption isotherms of HPH-ZIF-8 at different reaction times. (d) The yields of HPH-ZIF-8 at different reaction times. Note: the molar ratio of Zn^{2+}:2-methylimidazole is 1:40. All the error bars in this figure represent the standard deviation (n = 3 independent experiments), data are presented as mean values ± SD.

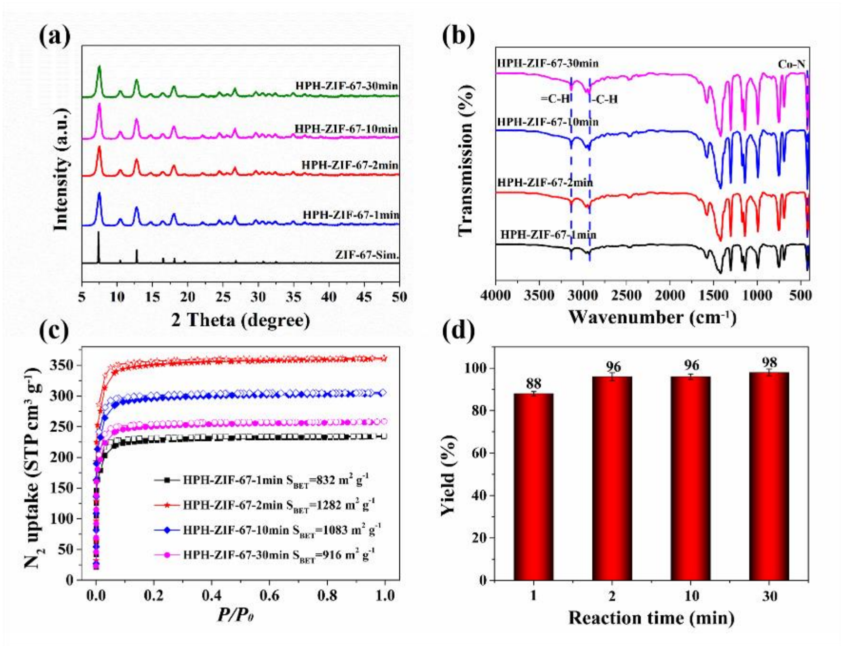

Supplementary Fig. 38. (a) PXRD patterns of HPH-ZIF-67 at different reaction times. (b) FT-IR spectra of HPH-ZIF-67 at different reaction times, several peaks located at ~3132, 2337 cm^{-1} and 424 cm^{-1} can be attributed to the =C-H, $-CH_3$ and Co–N[10, 11]. (c) N_2 sorption isotherms of HPH-ZIF-67 at different reaction times. (d) The yields of HPH-ZIF-67 at different reaction times. Note: the molar ratio of Co^{2+}:2-methylimidazole is 1:35. All the error bars in this figure represent the standard deviation (n = 3 independent experiments), data are presented as mean values ± SD.

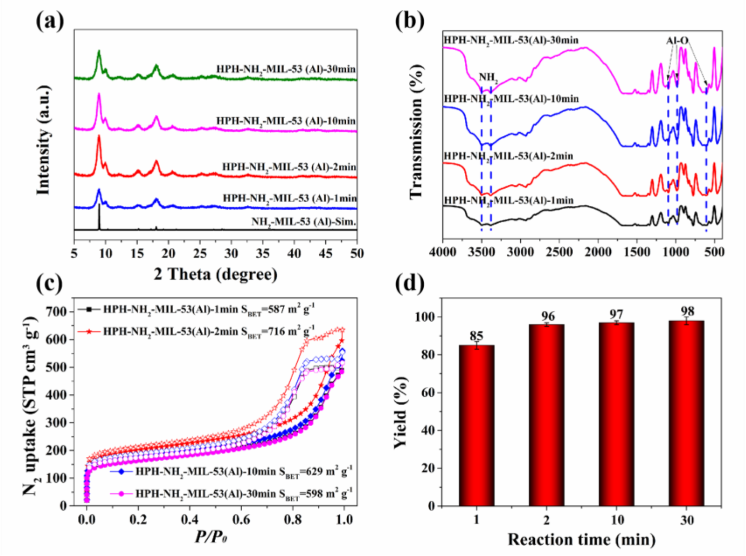

Supplementary Fig. 39. (a) PXRD patterns of HPH-NH_2-MIL-53(Al) at different reaction times. (b) FT-IR spectra of HPH-NH_2-MIL-53(Al) at different reaction times, several new peaks located at ~1095, 980 and 610 cm^{-1} can be attributed to the Al–O vibration peak, the peaks at 3380 and 3500 cm^{-1} were $-NH_2$ stretching vibration[12, 13]. (c) N_2 sorption isotherms of HPH-NH_2-MIL-53(Al) at different reaction times. (d) The yields of HPH-NH_2-MIL-53(Al) at different reaction times. All the error bars in this figure represent the standard deviation (n = 3 independent experiments), data are presented as mean values ± SD.

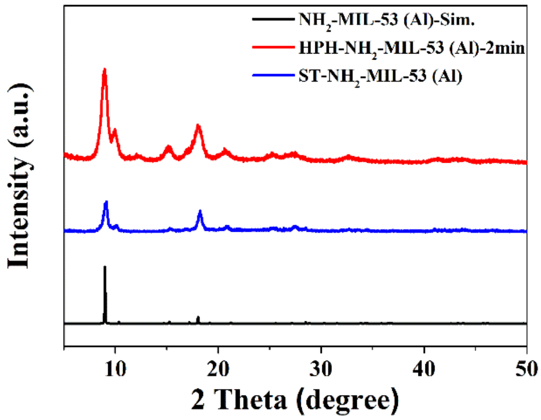

Supplementary Fig. 40. The comparison of PXRD patterns of HPH-NH_2-MIL-53(Al)-2min, ST-NH_2-MIL-53(Al), and simulated NH_2-MIL-53(Al). The PXRD peaks of HPH-NH_2-MIL-53(Al)-2min are in agreement with the reported ST-NH_2-MIL-53(Al)[14,15] (NH_2-MIL-53(Al) syntheiszed by solvothermal approach), which indicated the successful synthesize of HPH-NH_2-MIL-53(Al). Note: simulated PXRD was obtained based on the reference 15.

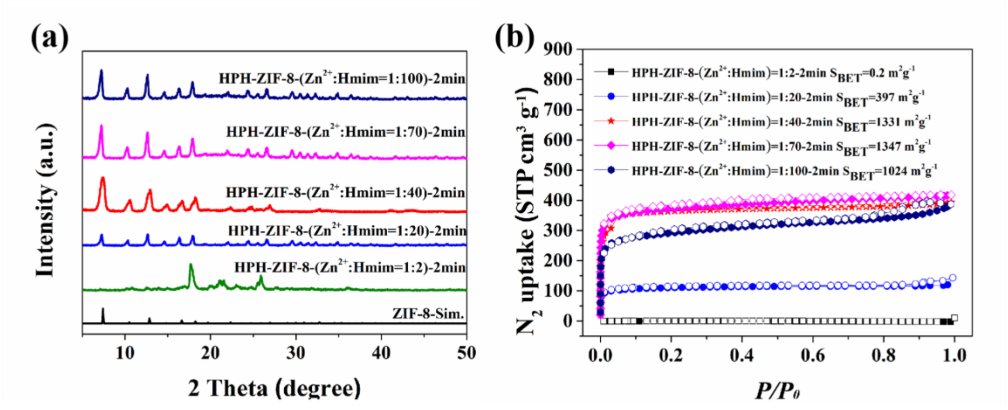

Supplementary Fig. 41. (a) PXRD patterns of HPH-ZIF-8-2min under different stoichiometric ratios. (b) N_2 sorption isotherms of HPH-ZIF-8-2min under different stoichiometric ratios.

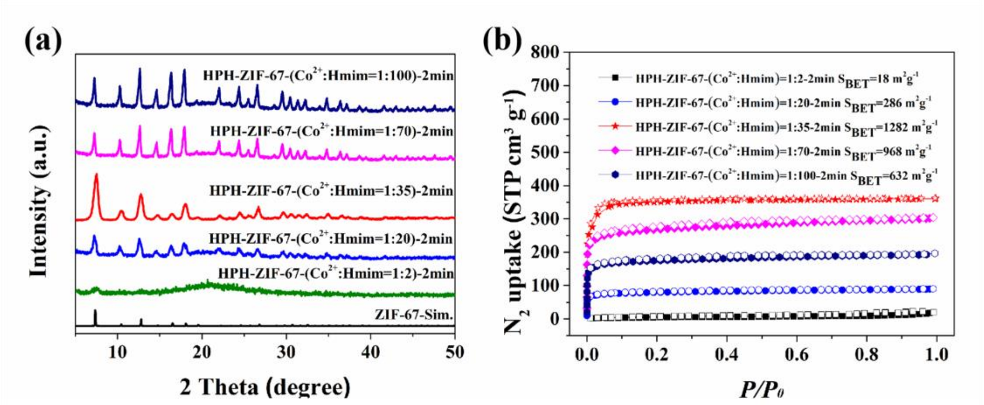

Supplementary Fig. 42. (a) PXRD patterns of HPH-ZIF-67-2min under different stoichiometric ratios. (b) N_2 sorption isotherms of HPH-ZIF-67-2min under different stoichiometric ratios.

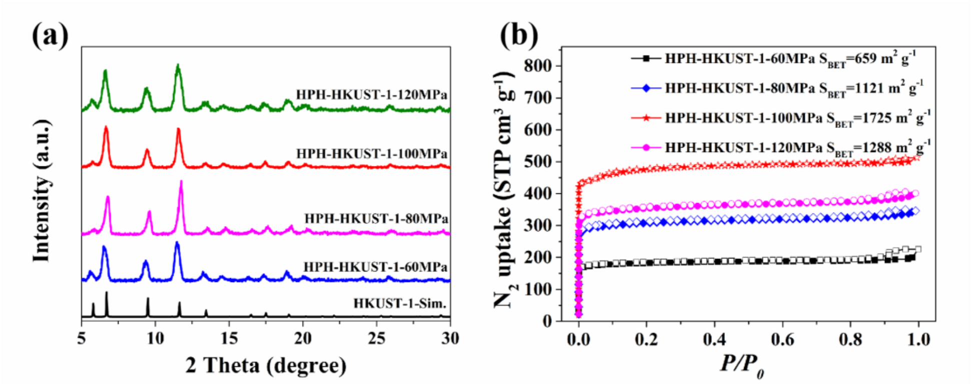

Supplementary Fig. 43. (a) PXRD patterns of HPH-HKUST-1 obtained by high pressure homogenization under different homogenization pressures for 2 min obtained by high pressure homogenization under different homogenization pressures for 2 min. (b) N_2 sorption isotherms of HPH-HKUST-1 obtained by high pressure homogenization under different homogenization pressures for 2 min.

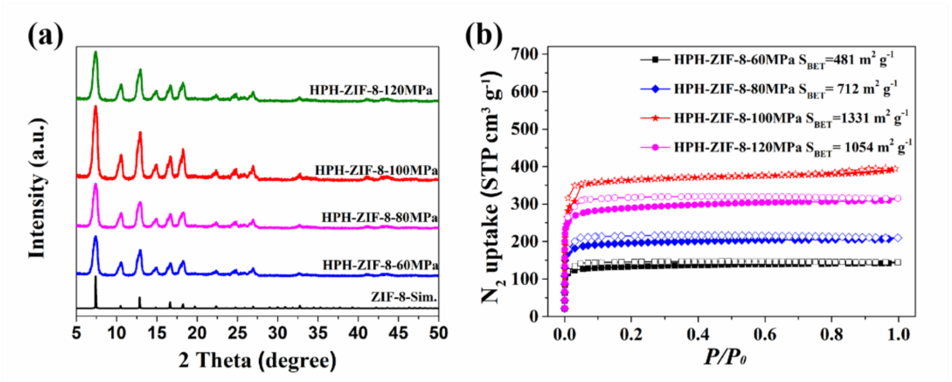

Supplementary Fig. 44. (a) PXRD patterns of HPH-ZIF-8 obtained by high pressure homogenization under different homogenization pressures for 2 min. (b) N_2 sorption isotherms of HPH-ZIF-8 obtained by high pressure homogenization under different homogenization pressures for 2 min. Note: the molar ratio of Zn^{2+}:2-methylimidazole is 1:40.

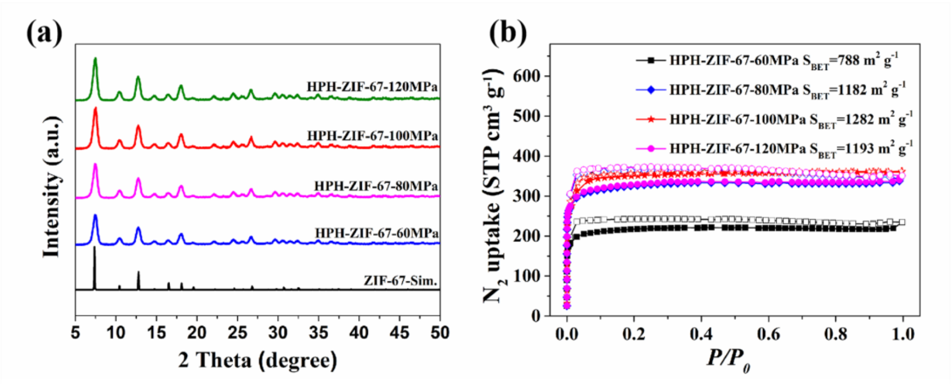

Supplementary Fig. 45. (a) PXRD patterns of HPH-ZIF-67 obtained by high pressure homogenization under different homogenization pressures for 2 min. (b) N_2 sorption isotherms of HPH-ZIF-67 obtained by high pressure homogenization under different homogenization pressures for 2 min. Note: the molar ratio of Co^{2+}:2-methylimidazole is 1:35.

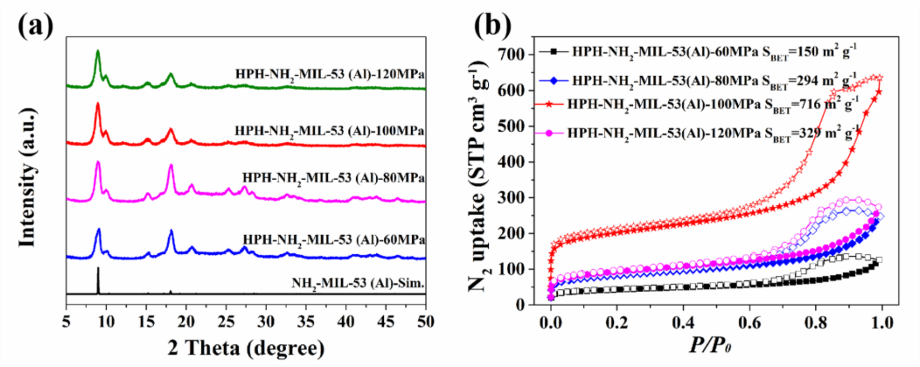

Supplementary Fig. 46. (a) PXRD patterns of HPH-NH_2-MIL-53-(Al) obtained by high pressure homogenization under different homogenization pressures for 2 min. (b) N_2 sorption isotherms of HPH-NH_2-MIL-53-(Al) obtained by high pressure homogenization under different homogenization pressures for 2 min.

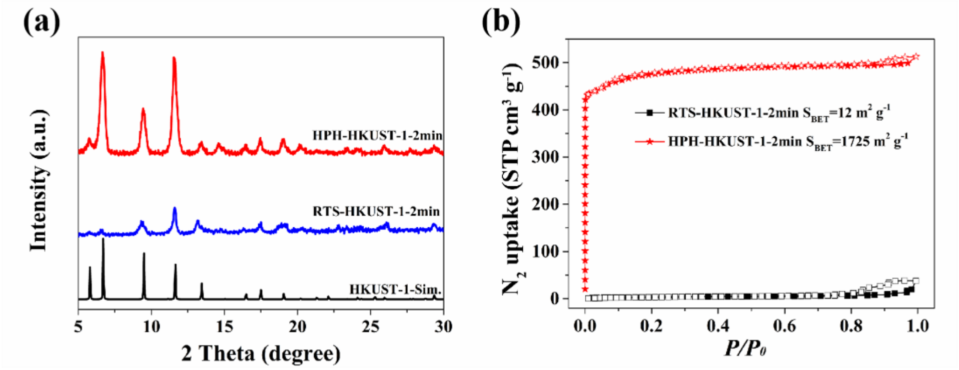

Supplementary Fig. 47. (a) PXRD patterns of HPH-HKUST-1 obtained by high pressure homogenization under 100 MPa for 2 min and HKUST-1 obtained via room temperature synthesis (RTS) with regular stir for 2 min. (b) N_2 sorption isotherms of HPH-HKUST-1 obtained by high pressure homogenization under 100 MPa for 2 min and HKUST-1 obtained via room temperature synthesis with regular stir for 2 min.

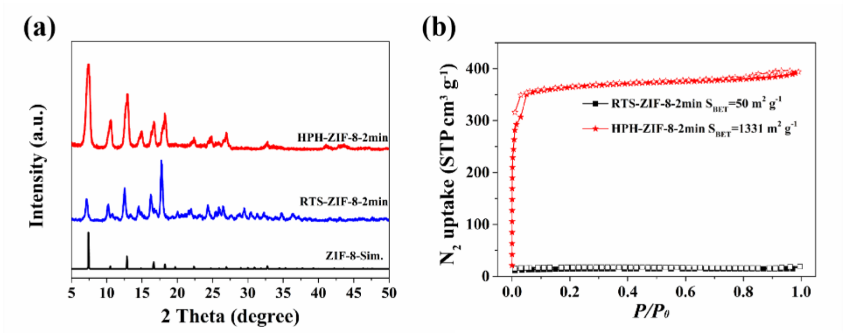

Supplementary Fig. 48. (a) PXRD patterns of HPH-ZIF-8 obtained by high pressure homogenization under 100 MPa for 2 min and ZIF-8 obtained via room temperature synthesis (RTS) with regular stir for 2 min. (b) N_2 sorption isotherms of HPH-ZIF-8 obtained by high pressure homogenization under 100 MPa for 2 min and ZIF-8 obtained via room temperature synthesis with regular stir for 2 min.

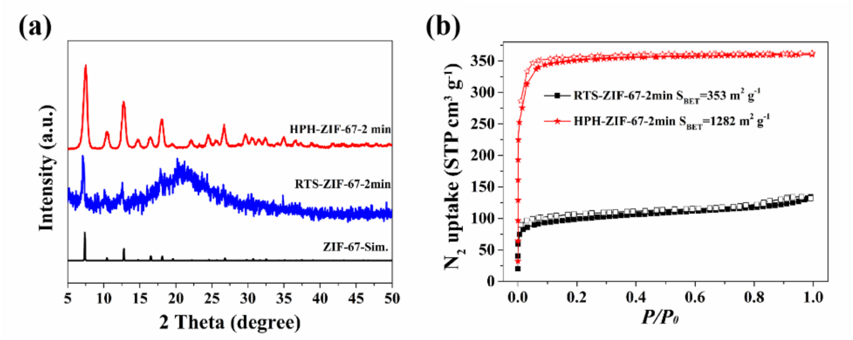

Supplementary Fig. 49. (a) PXRD patterns of HPH-ZIF-67 obtained by high pressure homogenization under 100 MPa for 2 min and ZIF-67 obtained via room temperature synthesis (RTS) with regular stir for 2 min. (b) N_2 sorption isotherms of HPH-ZIF-67 obtained by high pressure homogenization under 100 MPa for 2 min and ZIF-67 obtained via room temperature synthesis with regular stir for 2 min.

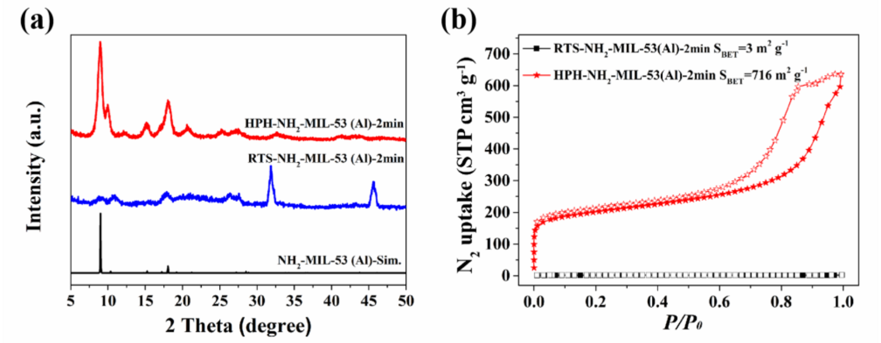

Supplementary Fig. 50. (a) PXRD patterns of HPH-NH_2-MIL-53(Al) obtained by high pressure homogenization under 100 MPa for 2 min and NH_2-MIL-53(Al) obtained via room temperature synthesis (RTS) with regular stir for 2 min. (b) N_2 sorption isotherms of HPH-NH_2-MIL-53(Al) obtained by high pressure homogenization under 100 MPa for 2 min and NH_2-MIL-53(Al) obtained via room temperature synthesis with regular stir for 2 min.

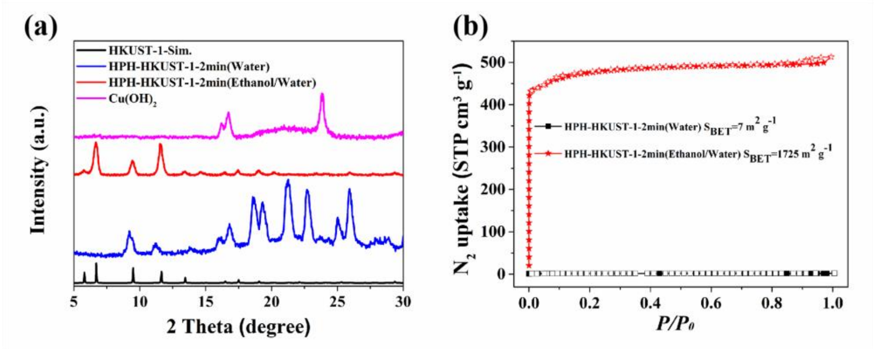

Supplementary Fig. 51. (a) PXRD patterns of $Cu(OH)_2$, HPH-HKUST-1(Water) and HPH-HKUST-1(Ethanol/Water) obtained by high pressure homogenization under 100 MPa for 10 min. (b) N_2 sorption isotherms of HPH-HKUST-1(Water) and HPH-HKUST-1(Ethanol/Water) obtained by high pressure homogenization under 100 MPa for 10 min.

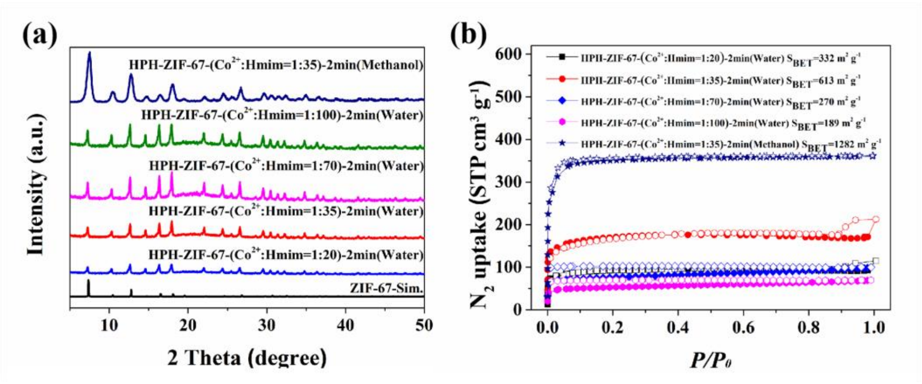

Supplementary Fig. 52. (a) PXRD patterns of HPH-ZIF-67(Methanol) and HPH-ZIF-67(Water) under different stoichiometric ratios obtained by high pressure homogenization under 100 MPa for 2 min. (b) N_2 sorption isotherms of HPH-ZIF-67(Methanol) and HPH-ZIF-67(Water) under different stoichiometric ratios obtained by high pressure homogenization under 100 MPa for 2 min.

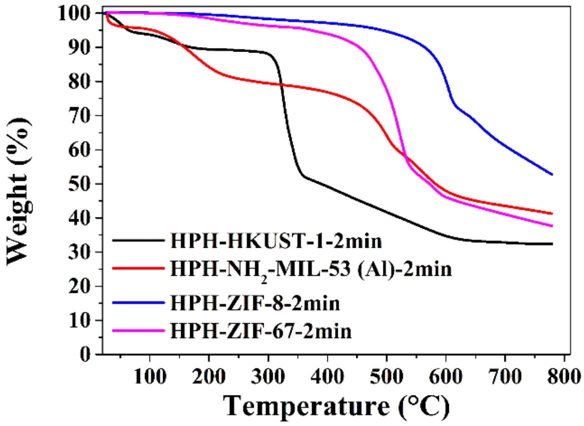

Supplementary Fig. 53. Thermogravimetric analysis of HPH-MOFs.

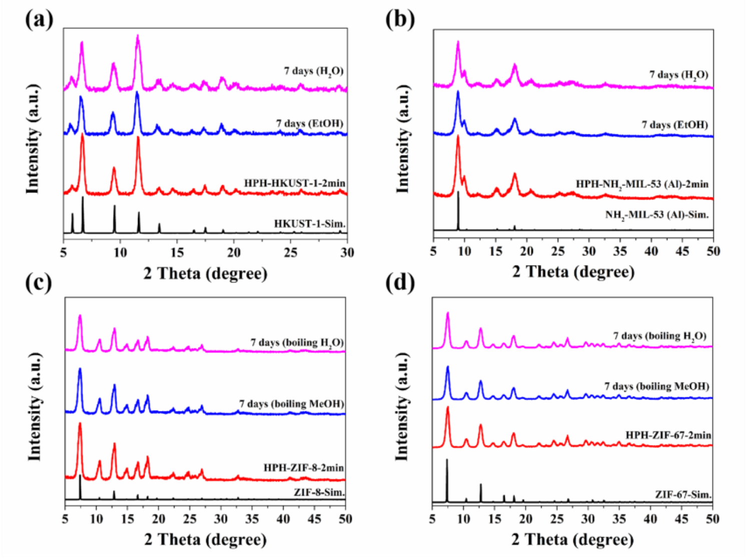

Supplementary Fig. 54. Solvent stability test for (a) HPH-HKUST-1-2min, (b) HPH-NH_2-MIL-53(Al)-2min, (c) HPH-ZIF-8-2min, (d) HPH-ZIF-67-2min.

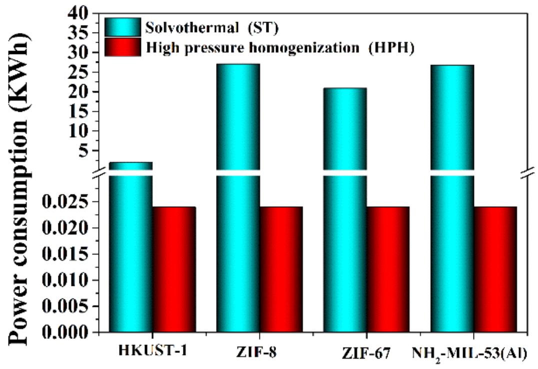

Supplementary Fig. 55. The comparison of power consumption of HPH-MOFs and ST-MOFs.

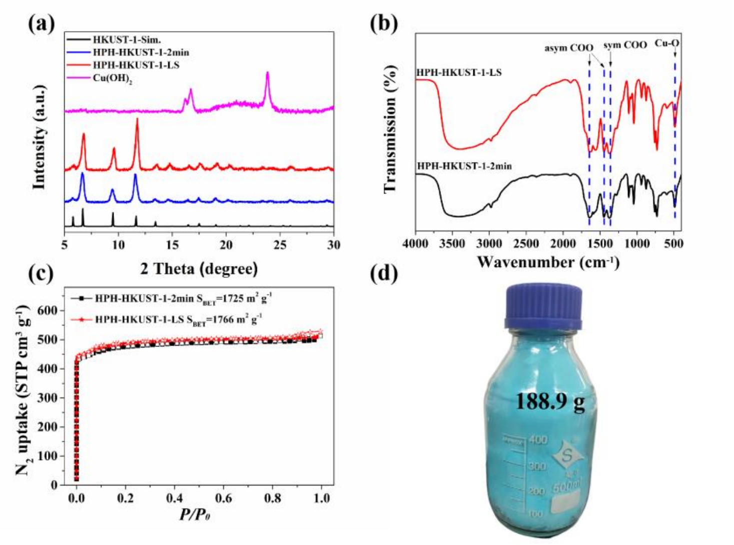

Supplementary Fig. 56. (a) The comparison of PXRD patterns of HPH-HKUST-1-2min, HPH-HKUST-1-LS (LS=Large-Scale), HKUST-1-simulated, and $Cu(OH)_2$. (b) The comparison of FT-IR spectra of HPH-HKUST-1-2min and HPH-HKUST-1-LS. (c) The comparison of N_2 sorption isotherms of HPH-HKUST-1-2min and HPH-HKUST-1-LS. (d) The photo of HPH-HKUST-1-LS obtained by high pressure homogenizer (discharge, 180 L h^{-1}).

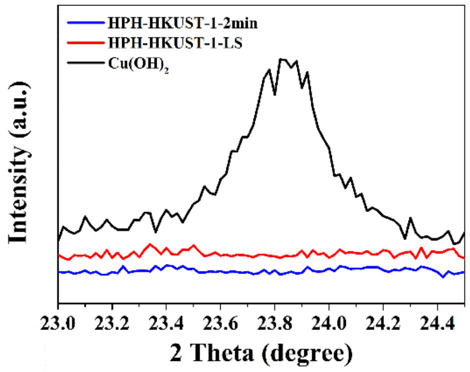

Supplementary Fig. 57. Crystallization evolution of HPH-HKUST-1-2min and HPH-HKUST-1-LS monitored by the disappear of PXRD peak in the range of 23–24.5° ($Cu(OH)_2$) for different materials.

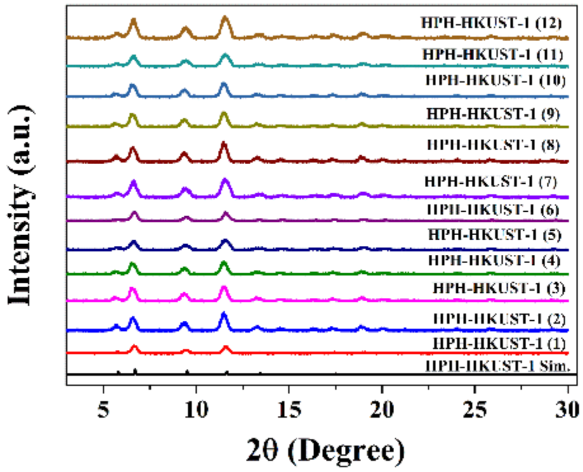

Supplementary Fig. 58. The PXRD of HPH-HKUST-1 obtained after solvent reused. HPH approach can realize the readily recyclability of solvent by adding 10 mL ethanol/water (2:1) after every six operations.

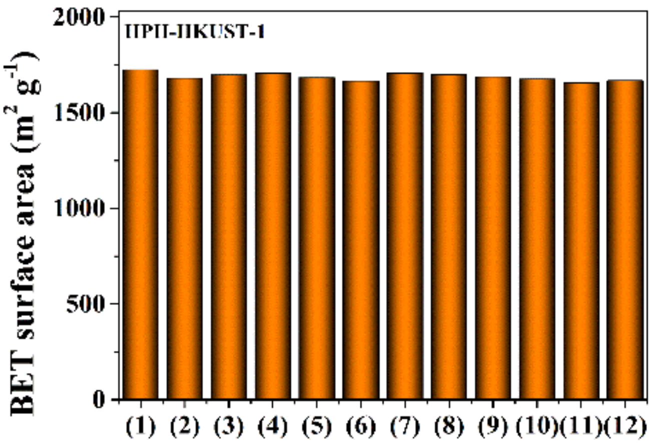

Supplementary Fig. 59. The BET surface area of HPH-HKUST-1 obtained after solvent reused. HPH approach can realize the readily recyclability of solvent by adding 10 mL ethanol/water (2:1) after every six operations.

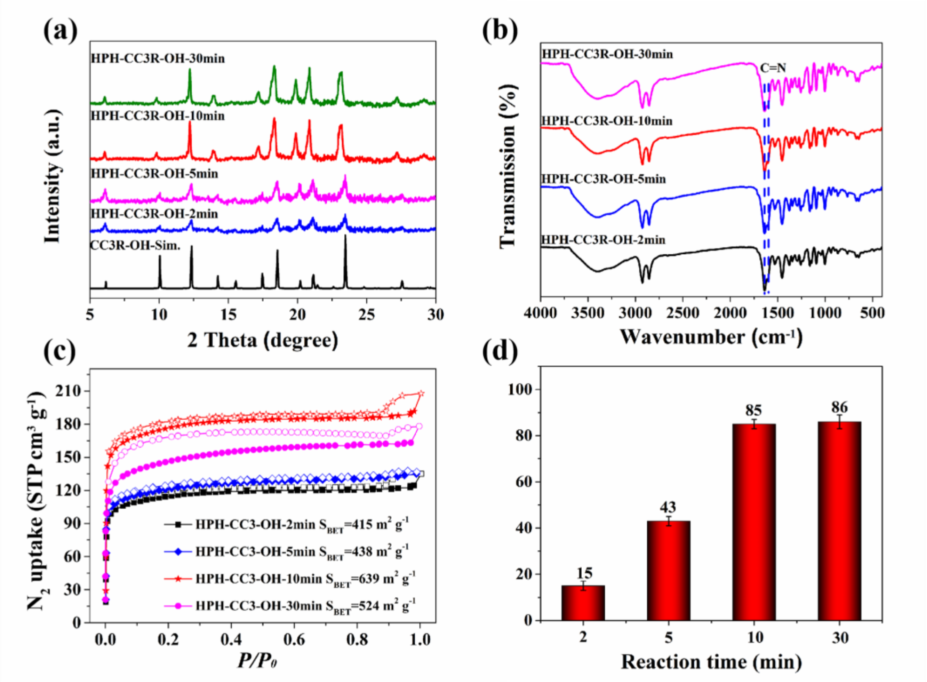

Supplementary Fig. 60. (a) PXRD patterns of HPH-CC3R-OH at different reaction times. (b) FT-IR spectra of HPH-CC3R-OH at different reaction times, several new imine stretching bands appeared at ~1634 and ~1601 cm^{-1} could be attributed to the C=N[16]. (c) N_2 sorption isotherms of HPH-CC3R-OH at different reaction times, (d) The yields of HPH-CC3R-OH at different reaction times. All the error bars in this figure represent the standard deviation (n = 3 independent experiments), data are presented as mean values ± SD.

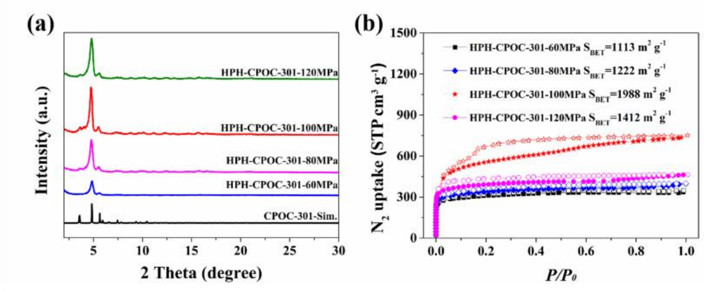

Supplementary Fig. 61. (a) PXRD patterns of HPH-CPOC-301 obtained by high pressure homogenization under different homogenization pressures for 2 min. (b) N_2 sorption isotherms of HPH-CPOC-301 obtained by high pressure homogenization under different homogenization pressures for 2 min.

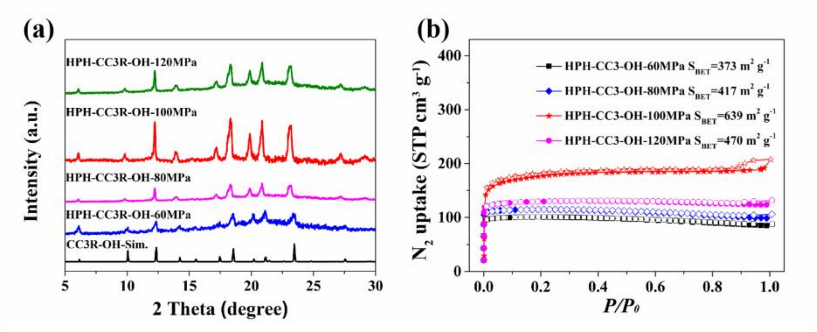

Supplementary Fig. 62. (a) PXRD patterns of HPH-CC3R-OH obtained by high pressure homogenization under different homogenization pressures for 10 min. (b) N_2 sorption isotherms of HPH-CC3R-OH obtained by high pressure homogenization under different homogenization pressures for 10 min.

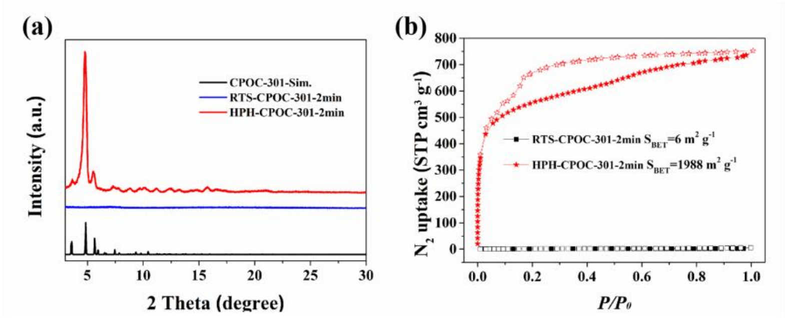

Supplementary Fig. 63. (a) PXRD patterns of HPH-CPOC-301 obtained by high pressure homogenization under 100 MPa for 2 min and CPOC-301 obtained via room temperature synthesis (RTS) with regular stir for 2 min. (b) N_2 sorption isotherms of HPH-CPOC-301 obtained by high pressure homogenization under 100 MPa for 2 min and CPOC-301 obtained via room temperature synthesis with regular stir for 2 min.

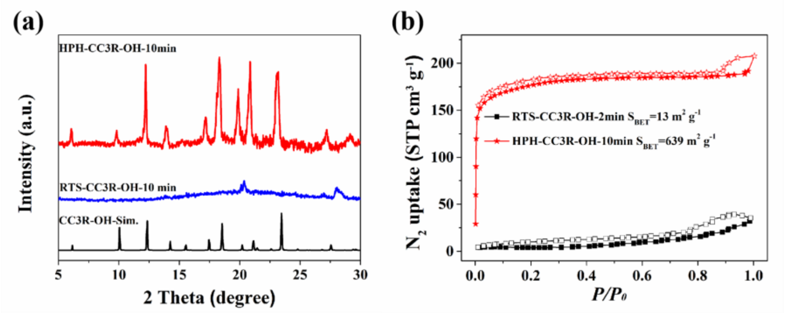

Supplementary Fig. 64. (a) PXRD patterns of HPH-CC3R-OH obtained by high pressure homogenization under 100 MPa for 10 min and CC3R-OH obtained via room temperature synthesis (RTS) with regular stir for 10 min. (b) N_2 sorption isotherms of HPH-CC3R-OH obtained by high pressure homogenization under 100 MPa for 10 min and CC3R-OH obtained via room temperature synthesis with regular stir for 10 min.

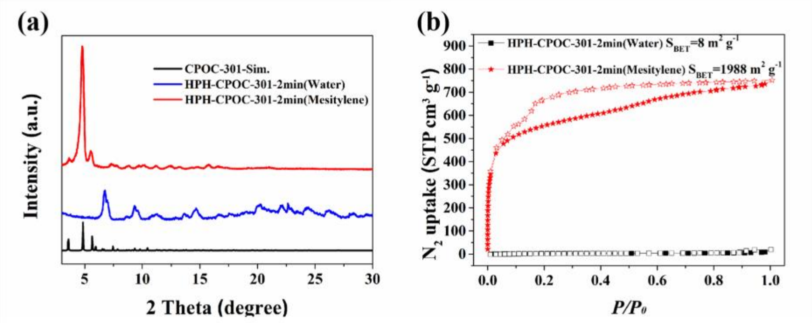

Supplementary Fig. 65. (a) PXRD patterns of HPH-CPOC-301(Water) and HPH-CPOC-301(Mesitylene) obtained by high pressure homogenization under 100 MPa for 2 min. (b) N_2 sorption isotherms of of HPH-CPOC-301(Water) and HPH-CPOC-301(Mesitylene) obtained by high pressure homogenization under 100 MPa for 2 min.

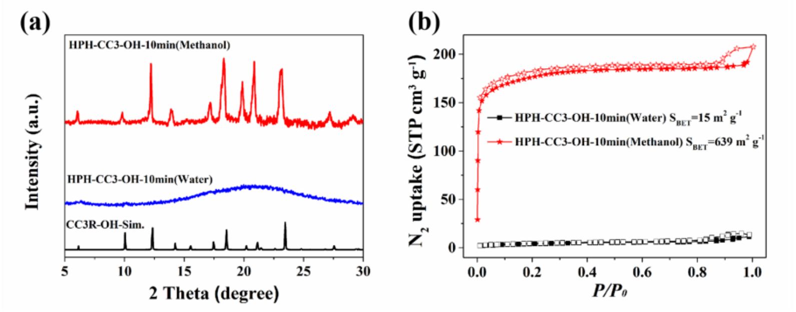

Supplementary Fig. 66. (a) PXRD patterns of HPH-CC3R-OH(Water) and HPH-CPOC-301(Methanol) obtained by high pressure homogenization under 100 MPa for 10 min. (b) N_2 sorption isotherms of HPH-CC3R-OH(Water) and HPH-CC3R-OH(Methanol) obtained by high pressure homogenization under 100 MPa for 10 min.

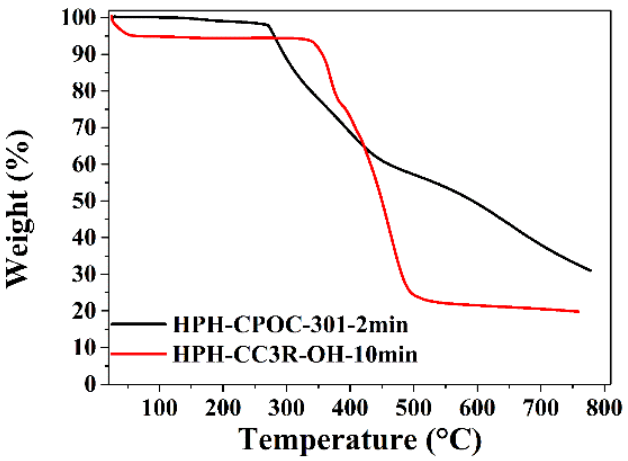

Supplementary Fig. 67. Thermogravimetric analysis of HPH-POCs.

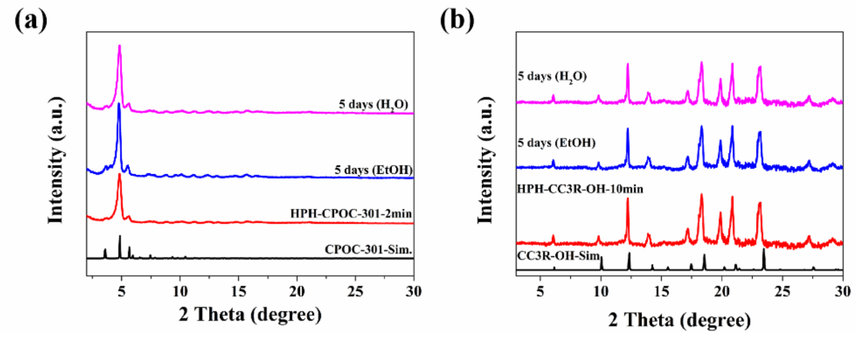

Supplementary Fig. 68. Solvent stability test for (a) HPH-CPOC-301-2min, and (b) HPH-CC3R-OH-10min.

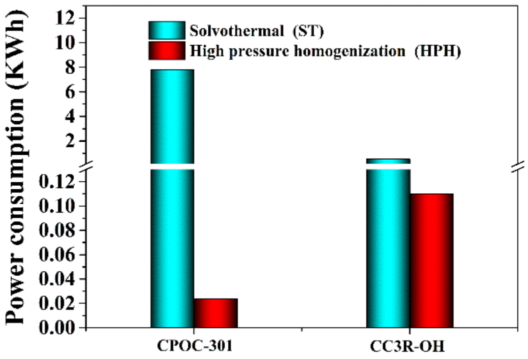

Supplementary Fig. 69. The comparison of power consumption of HPH-POCs and ST-POCs.

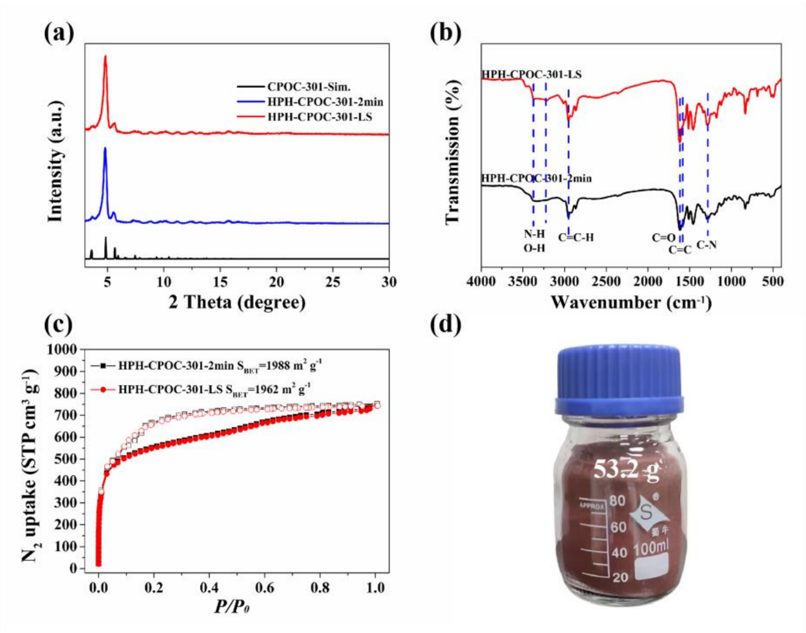

Supplementary Fig. 70. (a) The comparison of PXRD patterns of HPH-CPOC-301-2min and HPH-CPOC-301-LS (LS=Large-Scale). (b) The comparison of FT-IR spectra of HPH-CPOC-301-2min and HPH-CPOC-301-LS. (c) The comparison of N_2 sorption isotherms of HPH-CPOC-301-2min and HPH-CPOC-301-LS. (d) The photo of HPH-TpPa-1-LS obtained by high pressure homogenizer (discharge, 180 L h^{-1}).

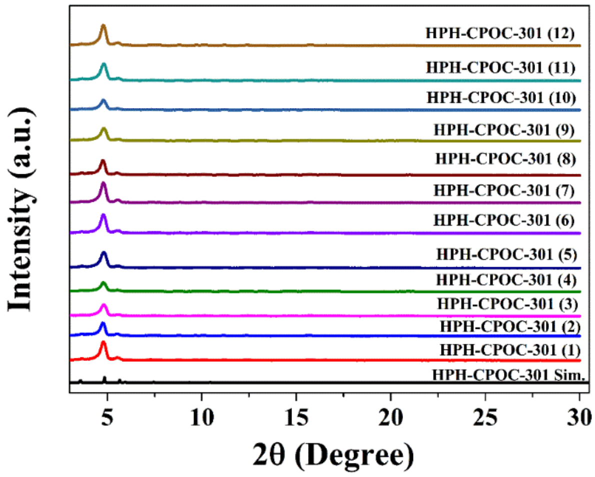

Supplementary Fig. 71. The PXRD of HPH-CPOC-301 obtained after solvent reused. HPH approach can realize the readily recyclability of solvent by adding 10 mL mesitylene after every six operations.

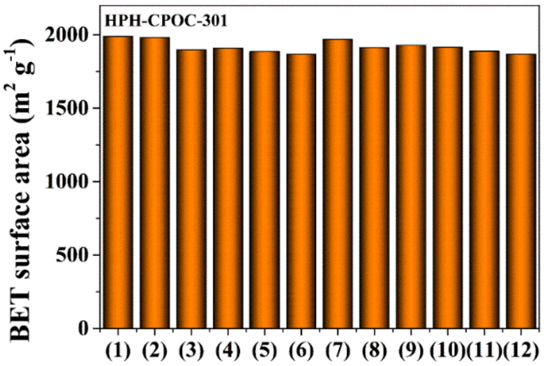

Supplementary Fig. 72. The BET surface area of HPH-CPOC-301 obtained after solvent reused. HPH approach can realize the readily recyclability of solvent by adding 10 mL mesitylene after every six operations.

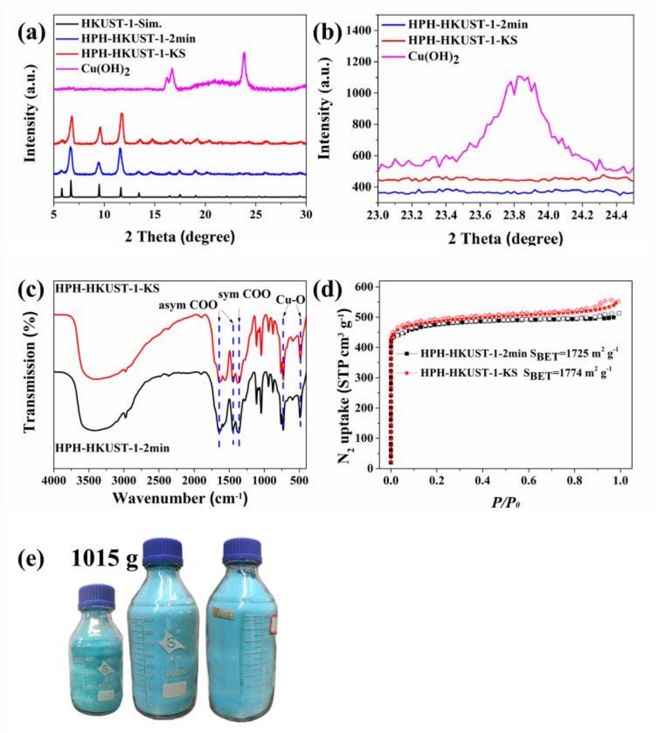

Supplementary Fig. 73. (a) The comparison of PXRD patterns of HKUST-1-simulated, HPH-HKUST-1-2min, HPH-HKUST-1-KS (KS=Kilogram-scale), and $Cu(OH)_2$. (b) Crystallization evolution of HPH-HKUST-1-2min and HPH-HKUST-1-KS monitored by the disappear of PXRD peak in the range of 23–24.5° ($Cu(OH)_2$) for different materials. (c) The comparison of FT-IR spectra of HPH-HKUST-1-2min and HPH-HKUST-1-KS. (d) The comparison of N_2 sorption isotherms of HPH-HKUST-1-2min and HPH-HKUST-1-KS. (e) The photo of HPH-HKUST-1-KS obtained by high pressure homogenizer (discharge, 180 L h^{-1}).

2.2 Supplementary Tables

Supplementary Table 1. The comparation of BET surface areas of different crystalline porous materials obtained by HPH approach and conventional room temperature synthesis (under same reaction time).

Sample	BET by HPH approach (m^2 g^{-1})	BET by room temperature synthesis approach (m^2 g^{-1})
TpPa-1	508	6
TpPa-2	312	4
TpBD	542	12
DAAQ	335	14
HKUST-1	1725	12
ZIF-8	1331	50
ZIF-67	1282	353
NH_2-MIL-53(Al)	716	3
CPOC-301	1988	6
CC3R-OH	639	13

Supplementary Table 2. The production rate of HPH-Laboratory and HPH-Industrial as well as Space-time-yield of the HPH-COFs, HPH-MOFs, and HPH-POCs.

Sample	Production rate of HPH-Laboratory Kg/day	Production rate of HPH-Industrial Ton/day	Space-time-yield kg m^{-3} day^{-1}
HPH-TpPa-1-30min	1.92	0.96	1.517×10^3
HPH-TpPa-2-30min	2.07	1.03	1.725×10^3
HPH-TpBD-30min	2.49	1.25	2.077×10^3
HPH-DAAQ-30min	2.89	1.44	2.381×10^3
HPH-HKUST-1-2min	897.54	448.77	4.4877×10^4
HPH-ZIF-8-2min	246.88	123.44	1.2344×10^4
HPH-ZIF-67-2min	450.84	225.42	2.2542×10^4
HPH-NH_2-MIL-53(Al)-2min	1160.96	580.48	5.8048×10^4
HPH-CPOC-301-2min	416.42	208.21	2.0821×10^4
HPH-CC3R-OH-10min	76.56	38.28	3.828×10^3

Note: HPH-Laboratory represent the high pressure homogenizer for laboratory use, and the running rate is 20 L h^{-1}; HPH-Factory represent the high pressure homogenizer for Factory use, the running rate is 10000 L h^{-1}.

Supplementary Table 3. The comparation of BET surface areas of some materials by HPH method using organic solvent or water as reaction medium.

Sample	BET by HPH approach using organic solvent (m^2 g^{-1})	BET by HPH approach using water (m^2 g^{-1})
HKUST-1	1725	7
ZIF-67	1282	613
CPOC-301	1988	8
CC3R-OH	639	15

Supplementary Table 4. The comparation of BET surface areas, Production rate, and Space-time-yield of different synthetic methods for MOFs and POCs.

Porous materials	Method	BET m^2 g^{-1}	Production rate Kg/day	Space-time-yield kg m^{-3} day^{-1}	Ref
HKUST-1	**High pressure homogenization**	**1725**	**4.4877×10^5**	**4.4877×10^4**	**This work**
	Commercially available named as Basolite C 300	1680	-	225	[17]
	Extrusion approach	1738	-	1.44×10^5	[18]
	Stirred at room temperature	1895	-	3.6×10^4	[6]
	Microfluidic synthesis	1911	0.0001	5.8	[19]
	Stirred at room temperature	1749	-	1842	[17]
	Aerosol synthesis	1470	-	494	[20]
	Electrochemical Synthesis	1441	-	-	[21]
	Solvothermal	692.2	-	-	[22]
	Solvothermal	1482	-	-	[23]
ZIF-8	**High pressure homogenization**	**1331**	**1.2344×10^5**	**1.2344×10^4**	**This work**
	Commercially available as Basolite Z1200	1925	-	100	[24, 25]
	Microreactor synthesis	1835	0.64	2.1×10^5	[26]
	Extrusion approach	1603	96	1.44×10^5	[18]
	Aerosol synthesis	1650	0.02448	69.3	[20]
	Room-Temperature Synthesis	962	-	-	[27]
	Solvothermal	1630	-	-	[28]
	Steam-assisted conversion	1470	-	-	[29]
	Sonochemical	1300	-	-	[30]
	ball mill	1480	-	-	[31]
ZIF-67	**High pressure**	**1282**	**2.2542×10^5**	**2.2542×10^4**	**This**

	**homogenization**				**work**
	Microfluidic synthesis	1258	6.84	5.7×10^{4}	10
	Scalable green synthesis	-	-	1.48×10^{3}	32
	Stirred at room temperature	1353	-	-	33
	Steam-assisted conversion method	1319	-	-	29
	Hydrothermal synthesis	316	-	-	34
NH_2-MIL-53(Al)	**High pressure homogenization**	**716**	**5.8048×10^{5}**	**5.8048×10^{4}**	**This work**
	Electrochemical Synthesis	788	-	-	21
	Solvothermal	675	-	-	14
CPOC-301	**High pressure homogenization**	**1988**	**2.0821×10^{5}**	**2.0821×10^{4}**	**This work**
	Solvothermal/vapor diffusion	1962	-	-	35
CC3R-OH	**High pressure homogenization**	**639**	**3.828×10^{4}**	**3.828×10^{3}**	**This work**
	Stirred and evaporated at room temperature	514	-	-	36
	Solvothermal	628.7	-	-	37

Supplementary Table 5. Comparison of advantages and disadvantages of different synthesis methods. The advantages of high pressure homogenization (HPH) technology in synthesizing crystalline porous materials including COFs, MOFs, and POCs compared with solvothermal (ST) method, mechanochemical (MC) synthesis, twin-screw extruder approach (TSE), microfluidic (MF) and microreactor (MR) synthesis. Note: RT represents room temperature, HT represents high temperature.

Method	Condition	Large scale	Mass and thermal transfer	Crystallinity	Surface area	Production rate	Continuous production	Cost
**HPH**	**Air/RT**	**Large**	**Good**	**High**	**High**	**High**	√	**Low**
ST	Air/HT/Vaccum	small	Good	High	High	Low	X	High
MC	Air/RT	Moderate	Poor	Low	Low	Moderate	X	Low
TSE	Air/HT	Moderate	Poor	High	High	Moderate	√	Low
MF	Air/RT	Low	Good	High	High	Low	√	Low
MR	Air/RT	Low	Good	High	High	Low	√	Low

Section 3 Supplementary References

1. Chong, J. H., Sauer, M., Patrick, B. O., MacLachlan, M. J. Highly stable keto-enamine salicylideneanilines. *Org. Lett.* **5**, 3823-3826 (2003).

2. Grajda, M., Wierzbicki, M., Cmoch, P., Szumna, A. Inherently chiral iminoresorcinarenes through regioselective unidirectional tautomerization. *J. Org. Chem.* **78**, 11597-11601 (2013).

3. Kandambeth, S., Mallick, A., Lukose, B., Mane, M. V., Heine, T., Banerjee, R. Construction of crystalline 2D covalent organic frameworks with remarkable chemical (acid/base) stability via a combined reversible and irreversible route. *J. Am. Chem. Soc.* **134**, 19524-19527 (2012).

4. Biswal, B. P., Chandra, S., Kandambeth, S., Lukose, B., Heine, T., Banerjee, R. Mechanochemical synthesis of chemically stable isoreticular covalent organic frameworks. *J. Am. Chem. Soc.* **135**, 5328-5331 (2013).

5. DeBlase, C. R., Silberstein, K. E., Truong, T. – T., Abruna, H. D., Dichtel, W. R. β-Ketoenamine-linked covalent organic frameworks capable of pseudocapacitive energy storage. *J. Am. Chem. Soc*. 135, 16821-16824 (2013).

6. Zhao, J. et al. Facile conversion of hydroxy double salts to metal-organic frameworks using metal oxide particles and atomic layer deposition thin-film templates. *J. Am. Chem. Soc.* **137**, 13756-13759 (2015).

7. Chandra, S. et al. Chemically stable multilayered covalent organic nanosheets from covalent organic frameworks via mechanical delamination. *J. Am. Chem. Soc.* **135**, 17853-17861 (2013).

8. Thote, J. et al. Constructing covalent organic frameworks in water via dynamic covalent bonding. *IUCrJ* **3**, 402-407 (2016).

9. Zhang, H., Zhao, M., Yang, Y., Lin, Y. S. Hydrolysis and condensation of ZIF-8 in water. *Micropor. Mesopor. Mater.* **288**, 109568 (2019).

10. Zhang, M. et al. Continuous synthesis of ZIF-67 by a microchannel mixer: a recyclable approach. *Micropo. Mesopor. Mater.* **327**, 111423 (2021).

11. Liu, D. et al. MOFs decorated sugarcane catalytic filter for water purification. *Chem. Eng. J.* **431**, 133992 (2022).

12. Xu, S., Ni, Y. NH_2-MIL-53(Al) nanocrystals: a fluorescent probe for the fast detection of aromatic nitro-compounds and ions in aqueous systems. *Analyst* **144**, 1687-1695 (2019).

13. Li, C., Xiong, Z., Zhang, J., Wu, C. The strengthening role of the amino group in metal-organic framework MIL-53 (Al) for methylene blue and malachite green dye adsorption. *J. Chem. Eng. Data* **60**, 3414-3422 (2015).

14. Gascon, J., Aktay, U., Hernandez-Alonso, M. D., van, Klink, G. P. M., Kapteijn, F. Amino-based metal-organic frameworks as stable, highly active basic catalysts. *J. Catal.* **261**, 75-87 (2009).

15. Couck, S., Denayer, J. M., Baron, G. V., Rémy, T, Gascon, J, Kapteijn, F. An amine-functionalized MIL-53 metal−organic framework with large separation power for CO_2 and CH_4. *J. Am. Chem. Soc*. **131**, 6326–6327 (2009).

16. Petryk, M. *et al.* Chiral, triformylphenol-derived salen-type [4 + 6] organic cages. *Org. Biomol. Chem.* **14**, 7495-7499 (2016).

17. Majano, G., Pérez-Ramírez, J. Scalable room-temperature conversion of copper(II) hydroxide into HKUST-1 ($Cu_3(btc)_2$). *Adv. Mater.* **25**, 1052-1057 (2013).

18. Crawford, D., Casaban, J., Haydon, R., Giri, N., McNally, T., James, S. L. Synthesis by extrusion: continuous, large-scale preparation of MOFs using little or no solvent. *Chem. Sci.* **6**, 1645-1649 (2015).

19. Faustini, M. et al. Microfluidic approach toward continuous and ultrafast synthesis of metal-organic framework crystals and hetero structures in confined microdroplets. *J. Am. Chem. Soc.* **135**, 14619-14626 (2013).

20. Garcia, Marquez, A. et al. Green scalable aerosol synthesis of porous metal-organic frameworks. *Chem. Commun.* **49**, 3848-3850 (2013).

21. Martinez, Joaristi, A., Juan-Alcañiz, J., Serra-Crespo, P., Kapteijn, F., Gascon, J. Electrochemical synthesis of some archetypical Zn^{2+}, Cu^{2+}, and Al^{3+} metal organic frameworks. *Cryst. Growth. Des.* **12**, 3489-3498 (2012).

22. Chui, S. S.-Y., Lo, S. M.-F., Charmant, J. P. H., Orpen, A. G., Williams, I. D. A chemically functionalizable nanoporous material $[Cu_3(TMA)_2(H_2O)_3]_n$. *Science* **283**, 1148-1150 (1999).

23. Liu, J. et al. Experimental and theoretical studies of gas adsorption in $Cu_3(BTC)_2$: an effective activation procedure. *J. Phys. Chem. C* **111**, 9305-9313 (2007).

24. Czaja, A. U., Trukhan, N., Müller, U. Industrial applications of metal-organic frameworks. *Chem. Soc. Rev.* **38**, 1284-1293 (2009).

25. Garcés-Polo, S. I., Villarroel-Rocha, J., Sapag, K., Korili, S. A., Gil, A. A comparative study of CO_2 diffusion from adsorption kinetic measurements on microporous materials at low pressures and temperatures. *Chem. Eng. J.* **302**, 278-286 (2016).

26. Polyzoidis, A., Altenburg, T., Schwarzer, M., Loebbecke, S., Kaskel, S. Continuous microreactor synthesis of ZIF-8 with high space-time-yield and tunable particle size. *Chem. Eng. J.* **283**, 971-977 (2016).

27. Cravillon, J., Münzer, S., Lohmeier, S.-J., Feldhoff, A., Huber, K., Wiebcke, M. Rapid room-temperature synthesis and characterization of nanocrystals of a prototypical zeolitic imidazolate framework. *Chem. Mater.* **21**, 1410-1412 (2009).

28. Park, K. S. et al. Exceptional chemical and thermal stability of zeolitic imidazolate frameworks. *Proc, Natl. Acad. Sci. U S A* **103**, 10186-10191 (2006).

29. Shi, Q., Chen, Z., Song, Z., Li, J., Dong, J. Synthesis of ZIF-8 and ZIF-67 by steam-assisted conversion and an investigation of their tribological behaviors. *Angew. Chem. Int. Ed.* **50**,

672-675 (2011).

30. Cho, H.-Y., Kim, J., Kim, S.-N., Ahn, W.-S. High yield 1-L scale synthesis of ZIF-8 via a sonochemical route. *Micropor. Mesopor. Mater.* **169**, 180-184 (2013).

31. Tanaka, S., Kida, K., Nagaoka, T., Ota, T., Miyake, Y. Mechanochemical dry conversion of zinc oxide to zeolitic imidazolate framework. *Chem. Commun.* **49**, 7884-7886 (2013).

32. Wu, C. et al. ZIF-derived Co/NCNTs as a superior catalyst for aromatic hydrocarbon resin hydrogenation: scalable green synthesis and insight into reaction mechanism. *Chem. Eng. J.* **443**, 136193 (2022).

33. Yang, J., Chen, H.-Q., Shi, N., Wang, T., Liu, J., Pan, W.-P. Porous carbon with uniformly distributed cobalt nanoparticles derived from ZIF-67 for efficient removal of vapor elemental mercury: a combined experimental and DFT study. *Chem. Eng. J.* **428**, 132095 (2022).

34. Qian, J., Sun, F., Qin, L. Hydrothermal synthesis of zeolitic imidazolate framework-67 (ZIF-67) nanocrystals. *Mater. Lett.* **82**, 220-223 (2012).

35. Su, K., Wang, W., Du, S., Ji, C., Zhou, M., Yuan, D. Reticular chemistry in the construction of porous organic cages. *J. Am. Chem. Soc.* **142**, 18060-18072 (2020).

36. Jiang, S. et al. Core-shell crystals of porous organic cages. *Angew. Chem. Int. Ed.* **57**, 11082 (2018).

37. Wang, Z.-M., Cui, Y.-Y., Yang, C.-X., Yan, X.-P. Porous organic nanocages CC3 and CC3-OH for chiral gas chromatography. *ACS Appl. Nano Mater.* **3**, 479-485 (2020).
